# Morphogenesis and Cell Fate Determination within the Adaxial Cell Equivalence Group of the Zebrafish Myotome

**DOI:** 10.1371/journal.pgen.1003014

**Published:** 2012-10-25

**Authors:** Mai E. Nguyen-Chi, Robert Bryson-Richardson, Carmen Sonntag, Thomas E. Hall, Abigail Gibson, Tamar Sztal, Wendy Chua, Thomas F. Schilling, Peter D. Currie

**Affiliations:** 1 Australian Regenerative Medicine Institute, Monash University, Clayton, Australia; 2 Department of Developmental and Cell Biology, University of California Irvine, Irvine, California, United States of America; University of Pennsylvania School of Medicine, United States of America

## Abstract

One of the central questions of developmental biology is how cells of equivalent potential—an equivalence group—come to adopt specific cellular fates. In this study we have used a combination of live imaging, single cell lineage analyses, and perturbation of specific signaling pathways to dissect the specification of the adaxial cells of the zebrafish embryo. We show that the adaxial cells are myogenic precursors that form a cell fate equivalence group of approximately 20 cells that consequently give rise to two distinct sub-types of muscle fibers: the superficial slow muscle fibers (SSFs) and muscle pioneer cells (MPs), distinguished by specific gene expression and cell behaviors. Using a combination of live imaging, retrospective and indicative fate mapping, and genetic studies, we show that MP and SSF precursors segregate at the beginning of segmentation and that they arise from distinct regions along the anterior-posterior (AP) and dorsal-ventral (DV) axes of the adaxial cell compartment. FGF signaling restricts MP cell fate in the anterior-most adaxial cells in each somite, while BMP signaling restricts this fate to the middle of the DV axis. Thus our results reveal that the synergistic actions of HH, FGF, and BMP signaling independently create a three-dimensional (3D) signaling milieu that coordinates cell fate within the adaxial cell equivalence group.

## Author Summary

How specific genes and signals act on initially identical cells to generate the different tissues of the body remains one of the central questions of developmental genetics. Zebrafish are a useful model system to tackle this question as the optically clear embryo allows direct imaging of forming tissues, tracking individual cells in a myriad of different genetic contexts. The zebrafish myotome, the compartment of the embryo that gives rise to skeletal muscle, is subdivided into a number of specific cell types—one of which, the adaxial cells, gives rise exclusively to muscle of the “slow twitch” class. The adaxial cells give rise to two types of slow muscle cell types, muscle pioneer cells and non-muscle pioneer slow cells, distinguished by gene expression and different cellular behaviours. In this study we use lineage tracing live imaging and the manipulation of distinct genetic pathways to demonstrate that the adaxial cells form a cell fate “equivalence group” that is specified using separate signaling pathways that operating in distinct dimensions.

## Introduction

The mechanisms that are utilised to generate individual cell types from a set of equivalently fated set of precursors remains a central experimental focus of developmental biology. Studies from invertebrate systems have defined the concept of an equivalence group, where small clusters of lineage related cells are determined by a combination of inductive and intrinsic signals to adopt individual fates [1]–[6]. This concept faces many difficulties when applied to complex three dimensional tissues such as those that typify vertebrate development, where the direct lineage relationships of many cells remain ill defined and the complicated morphogenesis of many tissues precludes definition of models of equivalence.

Zebrafish provides perhaps one of the most tractable contexts in which to examine concepts of cell fate determination in a vertebrate embryo, as a variety of lineage tracing techniques can be deployed in different genetic contexts in real time within an optically accessible embryo. One zebrafish lineage that has been examined in some detail is the embryonic myotome of zebrafish. As in all vertebrates, the majority of skeletal muscle in zebrafish forms from precursor cells present in the somites, which arise by segmentation of paraxial mesoderm in a rostral to caudal progression on either side of neural tube and notochord along the main body axis of the embryo. This process, referred to as myogenesis, gives rise to distinct slow and fast twitch muscle populations that differ in contraction speeds, metabolic activities and motoneuron innervation. In zebrafish, the location and origin of these two different cell populations are topographically separable [7], [8]. The early differentiating slow-muscle cells arise from a particular subset of presomitic mesodermal cells, termed the adaxial cells, which at the end of gastrulation align medially against the notochord [8]. These precursors initially adopt a pseudo epithelial morphology but shortly after their incorporation within the formed somite, undergo stereotypic morphogenetic cell shape changes, moving from their columnar shape to flatten and interleave, adopting a triangular shape, that upon further differentiation results in single adaxial cells extending from one somite boundary to the other. These cells collectively flatten medio-laterally to form a set of elongated myocytes that span the somite, positioned against the notochord [9].

Ultimately, adaxial cells give rise to two distinct sub-types of slow muscle fibers: the superficial slow-twitch muscle fibers (SSFs) and the muscle pioneer cells (MPs). SSFs and MPs possess distinct morphological, molecular and functional properties. After undergoing the initial morphogenetic cell shape changes described above, SSFs migrate from their notochord-associated midline position to traverse the entire extent of the forming myotome and come to lie at its most lateral surface. There, the SSF precursors complete their differentiation to form a monolayer of approximately 20 slow twitch muscle fibers. By contrast, MPs (2 to 6 per somite) do not migrate from the midline and are the first cells of the zebrafish myotome to differentiate, forming slow twitch muscle fibers immediately adjacent to the notochord [10]. All slow fibers express slow isoforms of myosin heavy-chain (SMyHC) as well as the homeodomain protein Prox1 and are mono nucleated cells [11]. MPs, in addition, express high levels of homeodomain-containing Engrailed proteins [12], [13]. By contrast to slow precursors, differentiating fast precursors originate from the lateral somite and fuse to form multinucleated fibers, subsequently to SSF migration, and are distinguished by their expression of fast MyHC. A subset of these fibers, known as medial fast fibers (MFFs) also expresses Engrailed at lower levels than MPs [14]. The timing of the fate determination of these distinct cell types has been examined by a rigorous *in vivo* transplantation assays. By interchanging slow and fast muscle precursors at specific points in their development it has been demonstrated that at the time of gastrulation, although slow and fast muscle precursors are already spatially segregated, they remain uncommitted to their individual fates until they have entered into the segmental plate. Furthermore, the subdivision of adaxial compartment in to MP and non MP cell fates occurs at a similar period of development, with MP becoming irreversibly fated within the posterior part of the segmental plate during early somite formation [7].

In vertebrates, the specification and differentiation of the somite into specific cell types is under the influence of inductive signals from the somite itself or those derived from the surrounding tissues (reviewed in [15], [16]). In the case of zebrafish myogenesis, by far the most well understood inductive signals controlling myogenesis are the Hedgehog (HH) family of secreted glycoproteins, which emanate from the embryonic midline. Numerous studies in the last two decades have demonstrated that HH is necessary and sufficient for induction of the slow twitch muscle fate. Indeed, analysis of loss of function mutants in HH pathway genes and the use of the HH pathway inhibitor cyclopamine have demonstrated that the timing and the level of HH signaling are critical for the formation of different muscle identities, including the MP cells, which require the highest level of HH signaling for their formation [14], [17]–[19]. However, even though HH over-expression can induce supernumerary MP cells, this is not sufficient to convert the dorsal and ventral extremes of the myotome into MP cells [20], [21] suggesting that other signals could induce MP in the midline region or repress MP differentiation in the dorsal and ventral muscle cells [21].

A further complication of these analyses is that they fail to explain how the symmetry of the adaxial cell compartment is initially broken to generate the dichotomy of MP and SFF fates within equivalent sets of cells. As the adaxial cells flank the notochord and floorplate, the source of HH peptide secretion, all adaxial cells would initially be exposed to the same level of secreted HH peptides. Hence, it is unclear how different levels of HH could act to generate the MP cell fate within a subset of adaxial cells and suggests that additional signals must influence adaxial cell fate. Recent studies have shed some light on the nature of other secreted signals that may act to influence muscle cell formation. Several studies have shown that manipulation of BMP signaling can alter MP number [21], [22]. Furthermore, Smad5, a downstream effector of BMP signaling has been shown to be activated in the dorsal and ventral adaxial cells and is absent within the central region of the somite [22], [23]. In addition, Smad binding sites have been shown to regulate activity of the *eng2a* promoter, the *eng* gene expressed the earliest within MP precursors [12], [21]–[23]. Collectively, these studies suggest that BMP signaling can influence the number of different cell types within the embryonic zebrafish myotome, but exactly how this is achieved has yet to be determined mechanistically.

In this study, we utilize a combination of live imaging, retrospective and indicative fate mapping, molecular and genetic studies to demonstrate that MP and SSF precursors arise from distinct regions along the anterior-posterior (AP) and dorsal-ventral (DV) axes of the adaxial cell compartment. Uniquely, this regionalization is controlled by the action of different signal transduction pathways that act specifically to direct specification in distinct axial dimensions. We demonstrate that the *sprouty4*-mediated inhibition of FGF signaling induces MP cell fate in the anterior-most adaxial cells in each somite and that *radar*-mediated BMP signaling restricts this fate to the middle of the DV axis. Our results indicate that HH, FGF and BMP signaling synergize to determine cell fate within the adaxial cell equivalence group.

## Results

### Superficial slow twitch muscle and muscle pioneer precursors arise from distinct locations within the adaxial compartment

**Figure 1:**
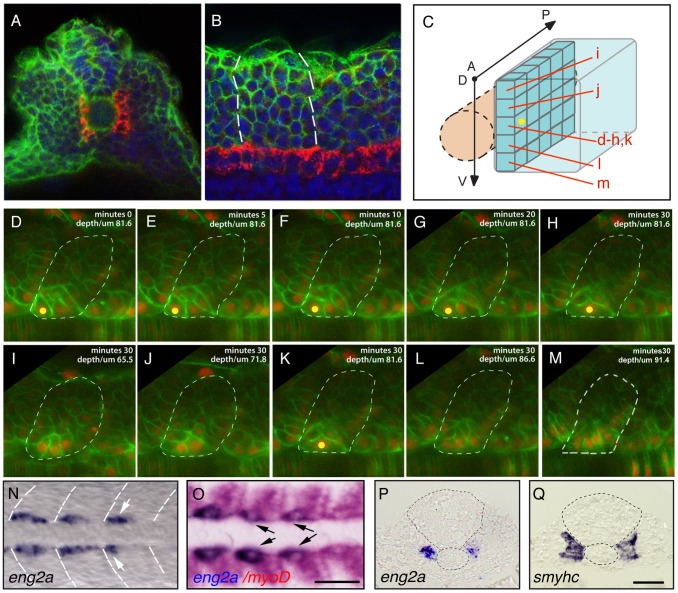
Distinct muscle precursor populations within the adaxial compartment. (A–C) The adaxial cell compartment. (A) Cross-section and (B) single confocal scan, dorsal view of a newly formed somite where the adaxial cells express the sMyHC (red). Nuclei and membranes are also marked with DAPI (blue) and membrane-bound GFP (green). (C–M) Restrospective fate map of the adaxial cell compartment. Adaxial cell behaviors occurring during the first phase of differentiation were analyzed in time lapse using a membrane-bound GFP (green) and a nuclear localized mCherry (red). The anterior-most adaxial cells in the dorso-ventral midline (yellow dot) are the first to differentiate and elongate. Adaxial cells above and below remain undifferentiated. The positions of individual confocal planes on the dorso-ventral axis are represented in (C). (N–Q) *eng2a* expression initiates in anterior adaxial cells at the dorso ventral midline of the myotome. 10-somite stage embryos on which *in situ* hybridization (ISH) for *eng2a* was performed alone (Blue, N, P) or in combination with *myoD* (Red, O,) that marks the posterior somitic region and the adaxial cells. Arrows indicate *eng2a* expression in the anterior adaxial cells. (Q) *In situ* hybridisation for *smyhc* demonstrates the location of the adaxial cells. (N, O) dorsal view, anterior toward the left, (P, Q) cross sections. Scale bar 50 µM.

In order to understand the origins of SSF and MP precursors from within the adaxial cell compartment (Figure 1A–1B), we examined adaxial cell behaviors during the first phase of their differentiation via continuous 4D time-lapse analysis and retrospective fate map analysis of the entire forming myotome. The position and shape of the adaxial cells were followed using a membrane-bound GFP and a nuclear localized mCherry whose expression in all cells was achieved after mRNA injection at 1-cell-stage. This analysis identified that the first adaxial cells to initiate differentiation and elongation arise adjacent to the anterior border of each somite at its DV mid-point (Figure 1C–1M and Video S1). These cells are most likely MPs, which previously have been shown to differentiate precociously [10]. To confirm this, we analyzed the expression of the MP marker gene *engrailed2a (eng2a)* during early somitogenesis by *in situ* hybridization. At the 10-somite stage *eng2a* transcripts were detected within newly formed somites exclusively within a subset of adaxial cells, adjacent to the anterior somitic border, located precisely at the mid-point of the DV axis of the somite (Figure 1N). To more precisely localize *eng2a* expression within the somite, we undertook dual *in situ* hybridization with *myod*, which marks the adaxial cells and the posterior aspect of the lateral somite, which contains the differentiating fast muscle progenitors (Figure 1O). This analysis confirmed that the expression of *eng2a* initiates specifically in the anterior-most cells of the newly formed somites. The positioning of cells initiating *eng2a* expression to the dorsal ventral midline of the forming myotome was confirmed in transverse sections of similarly staged embryos individually stained for *eng2a* and slow myosin heavy chain 1 (*smyhc1*) gene expression (Figure 1P, 1Q).

**Figure 2:**
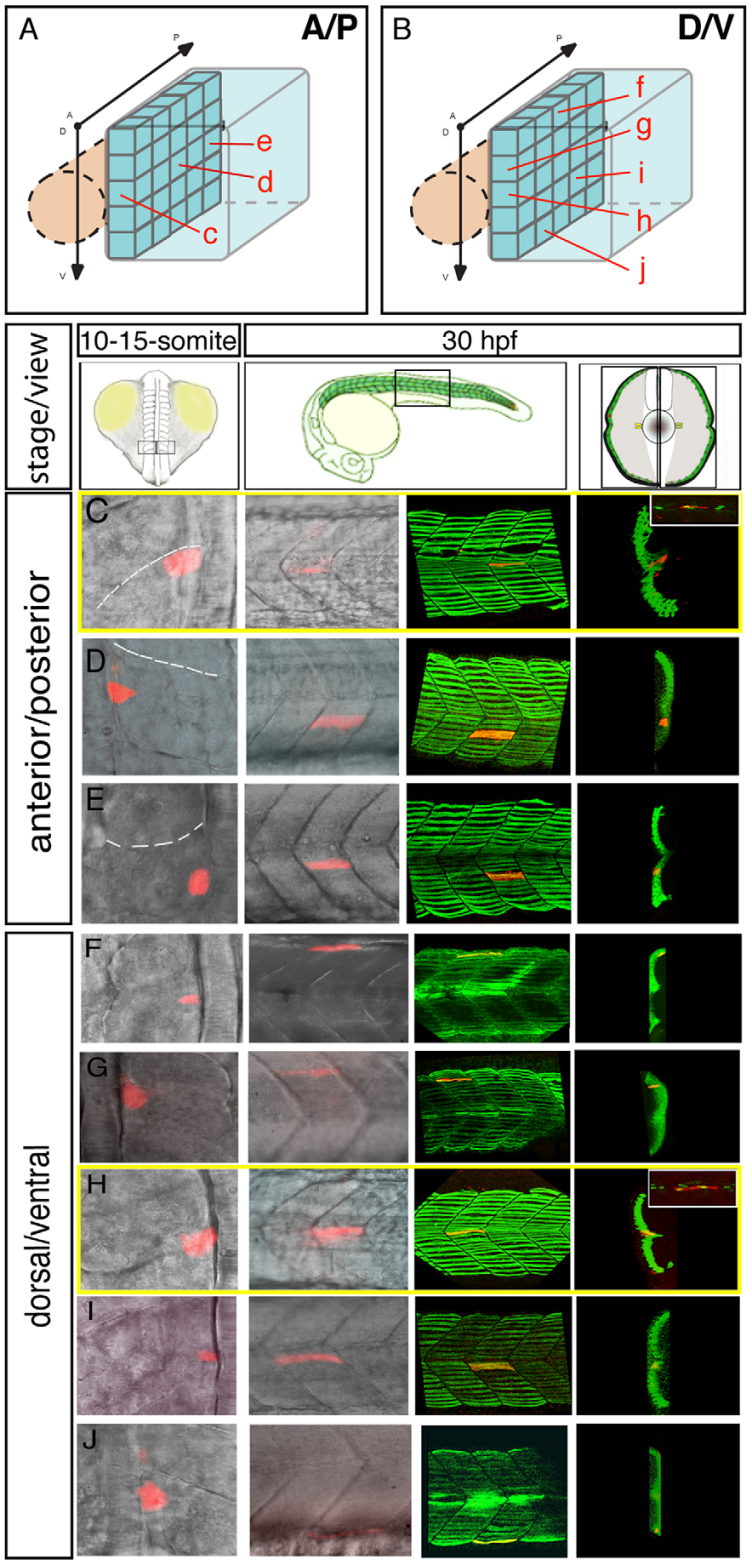
The fate map of the adaxial compartment. The fate of individual adaxial cells were identified using iontophoretic injections of TMRD (red) into individual adaxial cells located at various AP (C–E) and DV (F–J) positions within the three most newly formed somites of 10–15-somite stage embryos (C–J, left panels, dorsal views). The AP (A) and DV (B) position of labeled cells is represented schematically. The fate of cells was then analyzed at 30 hpf (C–J, other panels). (C–J) At 30 hpf, the panels (from the left to the right) represent the position of the labeled fiber in the myotome (lateral view), the expression of sMyHC (green, 3D reconstruction of multiple confocal scans) in lateral view and in cross section, and the expression of Engrailed (green, small upper panels for C and H, lateral view). MPs (C, H) were identified due to their position in the midline and simultaneous expression of Slow MyHC and Eng.

Collectively, these results suggest that SSF and MP precursors arise from distinct positions within the adaxial equivalence group. To test this hypothesis, we fate mapped the entire adaxial compartment by systematic iontophoretic injection of tetra-methyl rhodamine dextran (TMRD) lineage tracer dye into individual adaxial cells located at various AP and DV positions. Adaxial cells were labeled within the three most newly formed somites at the 10–15-somite stage and the fates of individually labeled cells were analyzed after the muscle fibers had terminally differentiated at 30 hpf. Individual injected embryos were sequentially incubated and imaged, first with an anti-Eng antibody and secondly with an anti-SMyHC antibody to unambiguously determine the fate of marked cells. This analysis confirmed that MP cells arise from the anterior-most adaxial cells at the dorso ventral midline of the somite (n = 8/8, Figure 2A, 2C, 2B, 2H) while posterior adaxial cells at this DV level make SSFs (n = 32/32, Figure 2A, 2D, 2E). Furthermore, we found that based on the initial position of a SSF precursor within the adaxial cell pool, we could predict its final location with the post migratory slow muscle palisade such that the dorsal- and ventral-most adaxial cells generate the dorsal and ventral-most post-migratory differentiated slow fibers respectively (n = 83, Figure 2B, 2F–2G, 2I–2J). This analysis not only demonstrates that MP and SSF precursors segregate at the beginning of somitogenesis but also determines the exact position of the precursors of every slow fiber. To further validate the fate of the adaxial cells located in the anterior somite at the DV mid-point, we examined their behaviour during the migration period. We thus performed a time-lapse analysis during a 20 hour period on embryos that were injected with a DNA construct containing the GFP gene under the control of the slow-twitch muscle-specific, *slow myosin heavy chain 1* (*smyhc1*) promoter. When located in the anterior margin of the somite, the transgenically labeled adaxial cell elongates in an anterior to posterior movement but remains adjacent to the notochord identifying the labelled cell as a MP (Video S2).

### FGF signaling controls the AP positioning of muscle pioneer precursors

**Figure 3:**
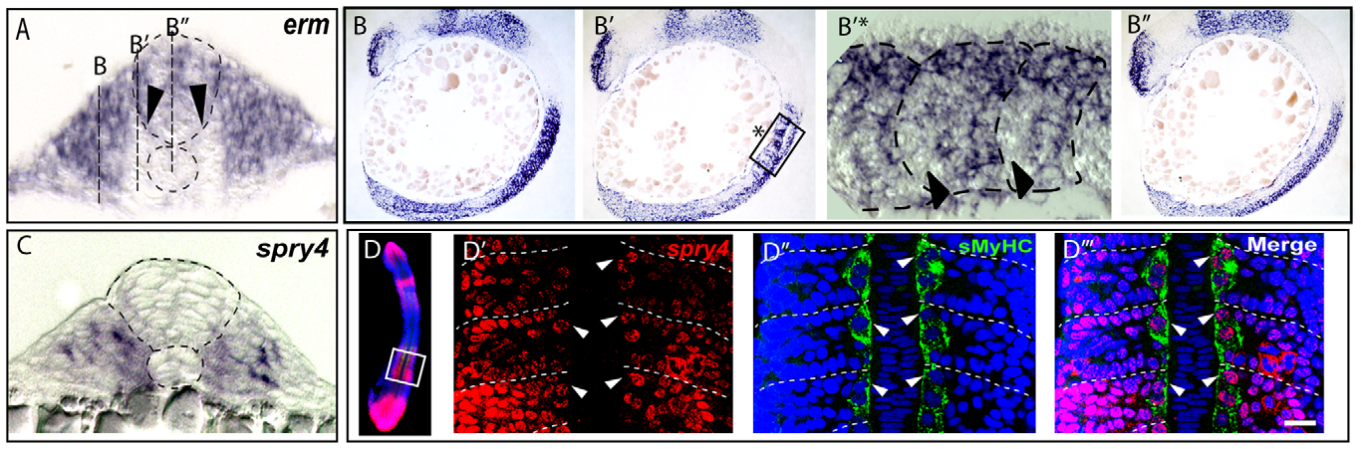
FGF signaling is down regulated in muscle pioneer precursor domain. (A–B) Asymmetric expression of *erm* at 9-somites as determined by ISH on (B–B″) serial sagittal sections and (A) cross-section. Panel (B′*) represents a high magnification of the region in box in (B′) panel. The different positions of sagittal sections are represented in (A). Expression of *erm* is absent in anterior adaxial cells (arrow heads). (C) *spry4* expression in a 9-somite embryo determined by ISH on a cross section of the anterior somitic region. (D–D‴) Expression of *spry4* mRNA (D′, red), sMyHC (D″, green) and DAPI (D–D‴, blue) in 9-somite embryos, dorsal view. *spry4* expression in the anterior adaxial cells is indicated (arrow heads). D′–D‴ high magnification view of the area boxed in D.

**Figure 4:**
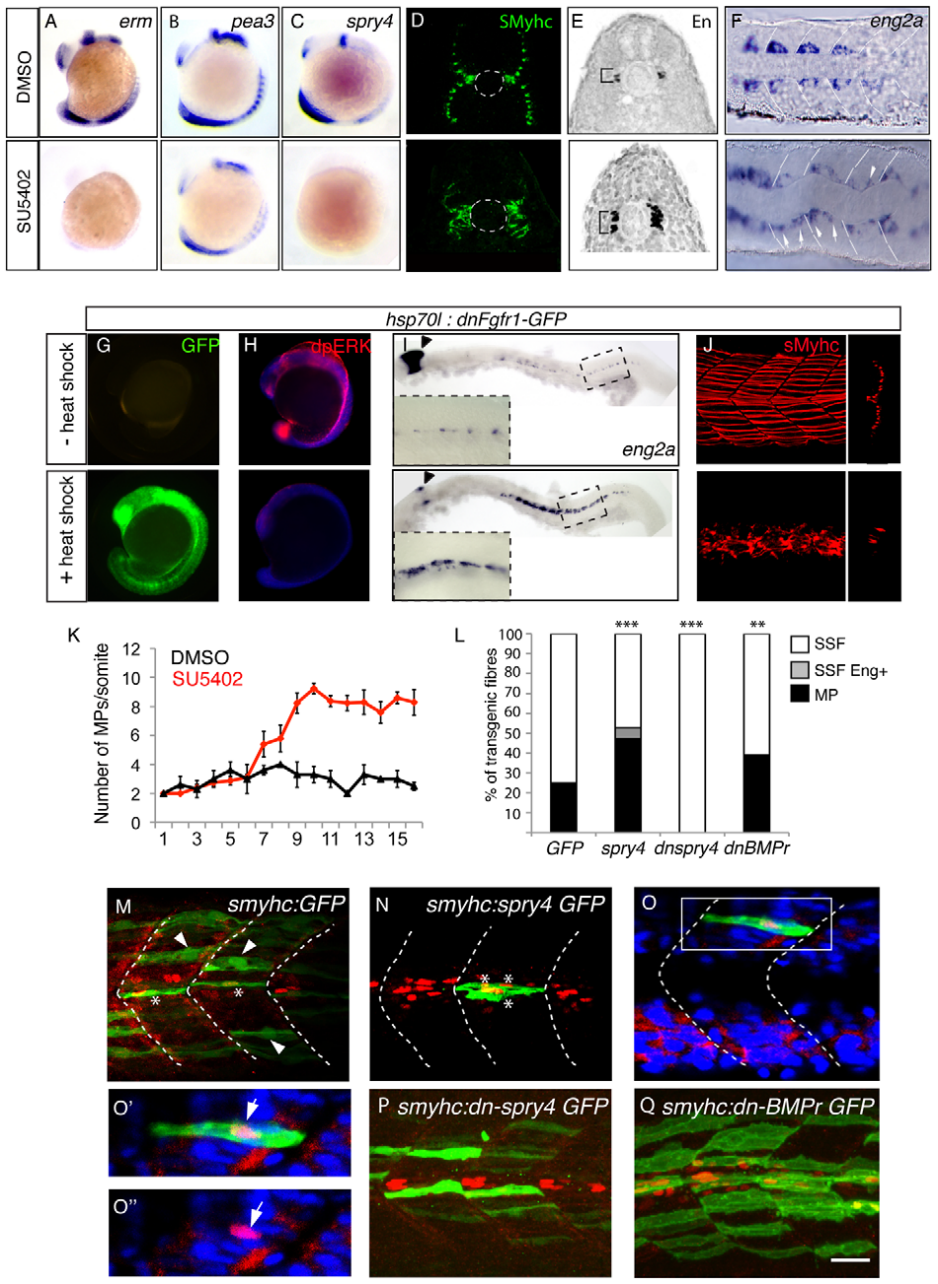
*sprouty4*-mediated FGF inhibition promotes muscle pioneer formation. *Erm* (A), *pea3* (B) and *spry4* (C) expressions in 10–13-somite embryos after DMSO or SU5402 treatments as determined by *in situ* hybridization. (D) sMyHC (green) and (E) Eng (black) expression in transverse sections of 1 dpf embryos, after treatment with DMSO or SU5402 applied at the 6-somite stage. (F) *eng2a* expression at 10-somite after treatment with DMSO or SU5402, as determined by ISH (Dorsal view, anterior towards the left). (G–J) Expression of a dominant negative form of fgfr1 fused with GFP (green) (G) is induced after heat shock in *hsp70l:dnfgfr1-GFP* embryos at 13–15-somite stage. (H) Expression of the target gene of FGF signaling cascade, diphosphorylated ERK (dpERK, red), which is down regulated in 13–15 somite embryos after heat shock compared to no heat shocked control. By contrast (I) *eng2a* is up-regulated at 1 dph after heat shock as revealed by ISH and (J) slow fibre migration is impaired, as revealed by 3D reconstructed sMyHC expression (lateral view and cross section). (K) Graphic representation of the number of MPs per somite in 1 dpf embryos after treatment with DMSO or SU5402 applied at the 10-somite stage. The horizontal axis represents the position of the somite along the body axis, 1 being the most rostral somite, values = means, error bars = standard error of the mean (s.e.m.). (L–Q) Ectopic expression of *spry4, dn-spry4* or *dn-BMPr* in slow precursors was obtained after injection of (L, N, O, O′, O″) *smyhc:spry4-ires-GFP*, (L, P) *smyhc:dn-spry4-ires-GFP and* (L, Q) *smyhc:dn-BMPr-GFP* DNA constructs into one cell embryo, respectively. (L, M) *smyhc:GFP* construct was used as the injection control. (L) Graphic representation of the different fates of transgenic slow fiber (SSF, MP and SSF en^+^) upon ectopic expression of *GFP, spry4*, *dnspry4* or *dnBMPr.* Values = means of percentages of transgenic muscle fibers per injected embryo (n_embryo_ = 6–9 per condition). We performed analysis of variance (ANOVA) to determine statistical difference within a 95% confidence interval: ** p<0.005, ***p<0.001. (M–Q) Images reveal the expression of GFP (green) and Eng (red) in the somites of 1 dpf embryos by maximum projections of multiple confocal scans (M, N, P, Q) or single confocal scan (O–O″). SSFs (arrow heads) and MPs (*) are indicated. (O–O″) In 4.19% of cases, the *spry4* transgene triggers expression of Engrailed in post-migratory slow fibers (arrows, p<0.05). (O′, O″) high magnification of the region boxed in O. Scale bar = 25 µm.

We next turned our attention to the molecular basis of the adaxial cell fate specification events that we had defined by our fate mapping strategies. A candidate approach, examining AP restricted inductive signals within the myotome, highlighted the FGF pathway as a putative regulator of AP patterning in the adaxial progenitors. Indeed, in zebrafish, at least two of the genes encoding *fgf* ligands, *fgf8* and *fgf17b* have been shown to be restricted in expression to the anterior somite [24], [25], [26]. However an analysis of the expression of the downstream targets of the FGF cascade, *erm* and *pea3* surprisingly revealed that asymmetric FGF responses occur specifically within the adaxial cells such that the anterior-most cells lose expression of FGF target genes during somite formation (Figure 3A, 3B–3B″ and data not shown). The temporal and spatial regulation of FGF signal activation during zebrafish myogenesis suggests a simple hypothesis. Distinct levels of FGF activation along the AP axis of the somite inform the adaxial cells of their position within this axis and consequently control their fate. In order to test this hypothesis we disrupted FGF signaling by the addition of the pharmacological inhibitor SU5402, a drug that blocks the phosphorylation of FGF receptors (FGFRs) and so prevents downstream signaling, as revealed by the downregulation of the target genes *erm*, *pea3* and *spry4* in SU5402 treated embryos (Figure 4A–4C, [25], [27]). SU5402 treatments at the 6-somite stage did not affect the number of slow muscle fibers (Table S1) but instead increased the number of MPs at the expense of SSFs, as revealed by a failure in slow-muscle fiber migration to the surface of the myotome and a corresponding increase in Engrailed positive MP cells evident at the midline (Figure 4D, 4E). Furthermore FGF inhibition does not alter the number of En positive medial fast fibers (Figure S1). The increase in MP number is foreshadowed by an expansion of the *eng2a* expression domain throughout the AP dimension of the adaxial cell compartment at the 10-somite stage (Figure 4F). Furthermore, the heat shock induced expression of a dominant negative form of FGFR1 that blocks the FGF/ERK signaling cascade also causes a similar increase of *eng2a* expression at the expense of SSF migration at 1 dpf (Figure 4G–4J). Collectively, these results show that FGF inhibition promotes the specification of the MP fate. Importantly, delayed addition of SU5402 until the 10-somite stage revealed that the more rostral 5–6 somites, which had already formed at the time of treatment, remained unaffected revealing a discrete temporal window of action for FGF signaling in MP specification within the newly formed somite (Figure 4K). This correlates specifically with the period of development when cuboidal cells are arrayed along the AP axis, prior to their differentiation (Figure 1A–1B). These data shows that FGF signaling inhibition specifies anterior identity and consequently MP fate within the adaxial cell equivalence group.

### FGF signaling patterns the adaxial cells independently from HH signaling

As described above the adaxial cells, and thus the slow twitch muscle lineage are highly dependent on Hedgehog (HH) signaling with the MP fate requiring higher levels and longer exposure to HH for proper specification than SSFs [14]. To test a possible cross talk between FGF and HH signaling, we analyzed the expression of HH target gene *patched1* (*ptc1*). However *ptc1* expression remains unaffected by SU5402 treatment (Figure S2A). FGF signaling was also recently shown to control the length of motile cilia within Kupffer's vesicle [28]. Although non-motile cilia are a distinct class of cell organelle, one possible mechanism for FGF action could be to regulate HH signal reception through the length or number of primary cilia on adaxial cells, as reception and activation of the HH pathway is controlled within the primary cilia in vertebrate cells [29]. However, our analysis suggests that SU5402 treatment doesn't affect the length or the number of primary cilia within the adaxial cells (Figure S2B). Therefore, the effect of FGF signaling on MP specification cannot be explained by modulation of HH transduction within adaxial cells.

### Sprouty4 controls muscle pioneer fate specification through FGF signal inhibition

To understand how the precise spatial activity of FGF is regulated to control the dichotomy of the cell fate decision evident with the adaxial cells, we systematically examined known inhibitors of the FGF pathway for their expression within the adaxial cells. This analysis revealed that *sprouty4* (*spry4*), which encodes a known intracellular inhibitor of receptor tyrosine kinases (RTKs), including the Fgfrs [30]–[33], becomes specifically activated in the anterior adaxial cells. Furthermore, the loss of expression of FGF target gene *erm* in the anterior adaxial cells correlates spatially and temporally with the induction of expression of the *spry4* gene in the identical cells (Figure 3C, 3D–3D‴). To test whether *spry4* expression influences MP and SSF fate specification, we ectopically expressed it within the adaxial cell compartment. Mosaic overexpression of *spry4* from the promoter of the *smyhc1* gene (*smyhc1:spry4-IRES-GFP*), which drives expression throughout the adaxial cell compartment [34], [35] doubled the number of MP cells (47.29% of transgenic fibres, n*_fibres_* = 143) within the embryo compared to control embryos expressing GFP alone (24.9%, n*_fibres_* = 521) (Figure 4L–4N). Furthermore, over-expression of *spry4* induces a third population of transgenic fibers that possess attributes of both MPs and SSFs. These rare fibers (4.19%, *n_fibres_* = 143) are able to migrate to the surface of the myotome and express Engrailed, a unique behavior never observed in control embryos (untreated or *smyhc:GFP* injected) (Figure 4L, 4O, 4O′ and 4O″). Reciprocally when we express a dominant negative form of *spry4*, using the identical *smyhc1* promoter (*smyhc1:dn-spry4-IRES-GFP*) cell autonomous loss of *spry4* leads to a loss of MP identity and adaxial cells that express *dnspry4* are incapable of making MPs (0% of transgenic fibres, n*_fibres_* = 48, Figure 4L, 4P, [36]).

**Figure 5:**
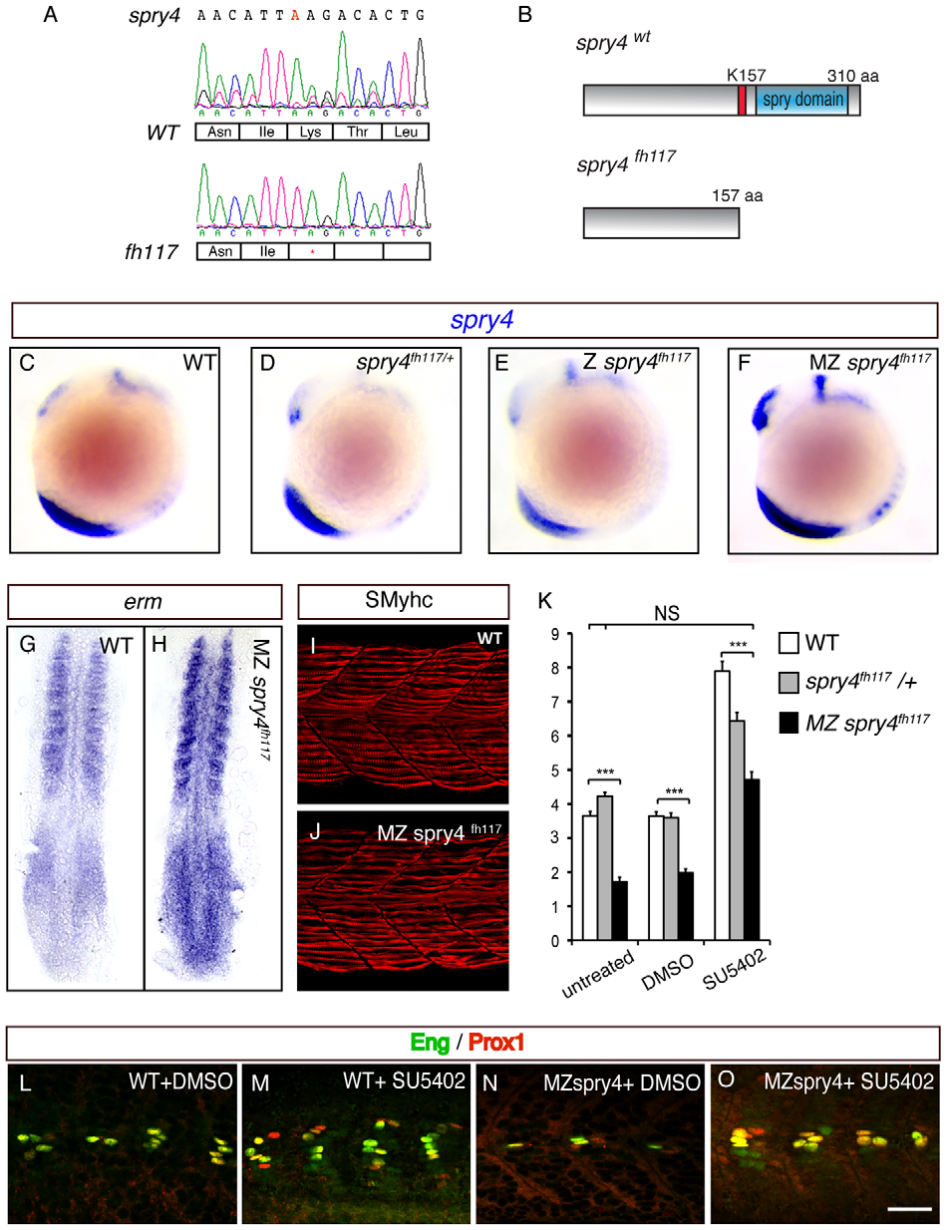
*sprouty4* loss-of-function leads to the reduction of MP formation. (A) Sequencing of *spry4^WT^* and *spry4^fh117^* confirms the single A to T mutation in position 469 of the *spry4* ORF, resulting in the premature stop codon. (B) Predicted peptide arising from *spry4^WT^* and *spry4^fh117^.* The mutation results in a truncated protein of 157 amino acids (K157 in red is replaced by a stop codon) lacking the conserved cysteine rich domain [31] also called the *spry* domain (blue). This domain has been involved in RTK inhibition in other systems. (C–F) *spry4* expression at 10-somite in WT, *spry4^fh117/+^*, Z *spry4^fh117^* and MZ *spry4^fh117^* embryos as determined by *in situ* hybridization. Whole mounted embryos, lateral view. (G–H) *Erm* expression in 9-somite WT and MZ *spry4^fh117^* embryos. Flat mounted embryos, anterior toward the top. (I–J) sMyhc expression (red) in the somites of (I) WT and (J) MZ *spry4^fh117^* embryos at 1 dpf. Maximal projection of multiple confocal scans, lateral view. (K) Graphic representation of the number of MPs per somite of 1 dpf embryos in different conditions: WT, *spry4^fh117^*/+, and MZ *spry4^fh117^* untreated embryos or after DMSO or SU5402 treatments, values = means, error bars = s.e.m, ***p<0.001. (L–O) Engrailed (green) and Prox1 (red) expression in the somites of 1 dpf WT or MZ *spry4^fh117^* embryos, after DMSO or SU5402 treatments, scale bar = 50 µm.

We next analyzed muscle development in mutants that have had the *spry4* gene inactivated. *spry4^fl117^* mutants carry a single A-to-T transversion, which introduces a stop codon early in the ORF of the gene (Figure 5A). The mutant allele encodes for a truncated protein, which lacks the putative activation domain involved in FGF signaling inhibition and is consequently predicted to engender a full loss of function in *spry4* (Figure 5B). Maternal zygotic (MZ) *spry4^fh117^* homozygous mutant embryos, but not heterozygous or zygotic (Z) mutants, exhibit a marked increase in FGF target gene expression (*erm*, *n* = 9/9; dpERK, *n* = 13/13; and *spry4*, *n* = 8/8, Figure 5C–5F and 5G–5H and data not shown), showing that FGF activity is increased in MZ *spry4^fh117^* mutants. Furthermore, while we could show that both the number of slow fibers and their position was unaffected in MZ *spry4^fh117^* mutant embryos (Figure 5I–5J, Table S1 and Figure S3) the number of MPs was less than half that of controls (n = 31, Figure 5K, 5L and 5N) a deficit that was rescued by SU5402 treatment (n = 32, Figure 5K, 5M and 5O), indicating that the deficiency of MPs associated with the loss of *spry4* is directly due to FGF over-activation and not modulation of other RTKs.

### Radar-mediated Bmp signaling coordinates MP and SSF fate specification synergistically with FGF signaling

**Figure 6:**
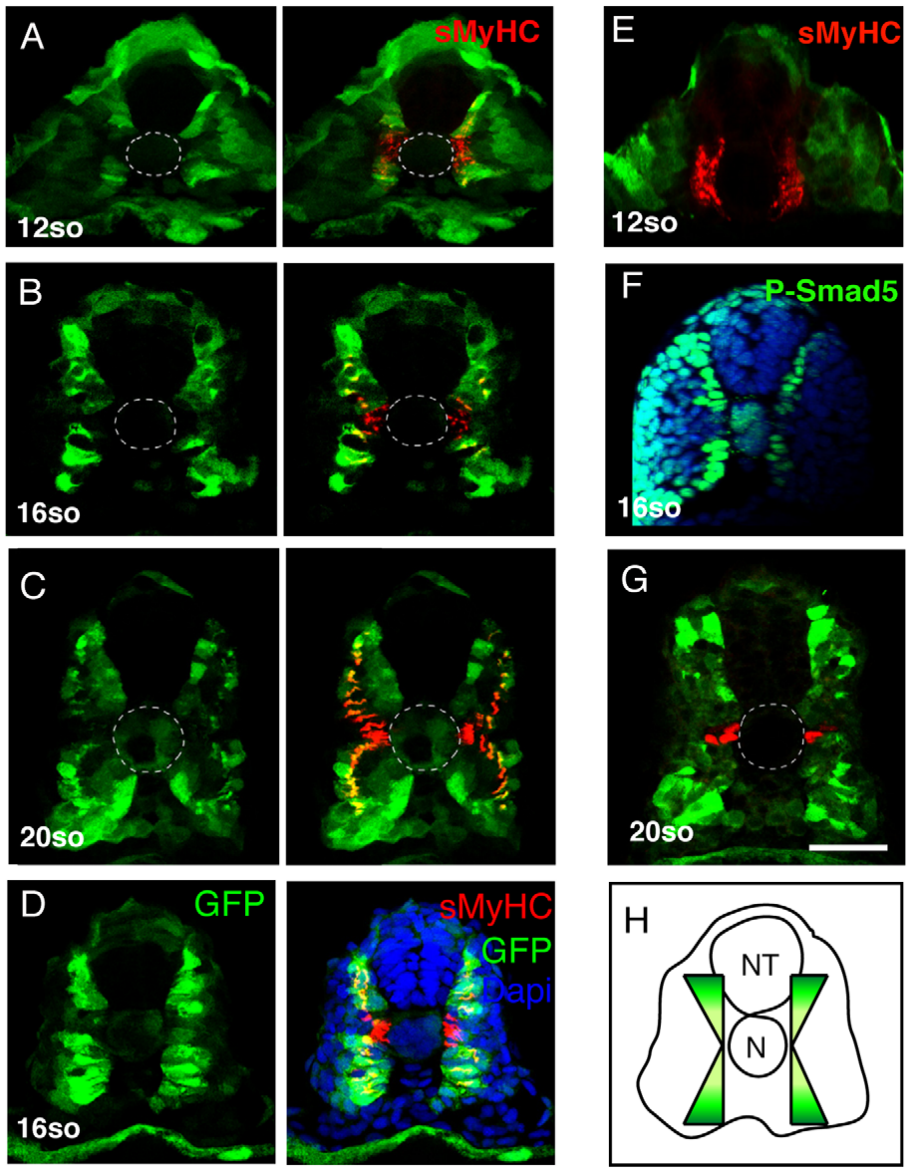
BMP signaling forms a dorsal and ventral gradient within the myotome. (A, B, C, D, E) Expression of GFP (green) and sMyHC (red) at indicated stages in Tg(*5XBre[vent2]:-201id3:gfp*) embryos uninjected or (E) after *radar* morpholino (*rdr^MO^*) injection. (F) Expression of phosphorylated-Smad5 (green) and nuclei (DAPI, blue) in 15-somite WT embryos. (G) Engrailed (red) and GFP (green) expression in Tg(*5XBre[vent2]:-201id3:gfp*) embryos at 20-somites. Cross-sections, maximum projections of multiple confocal scans. Scale bar 50 µm.

**Figure 7:**
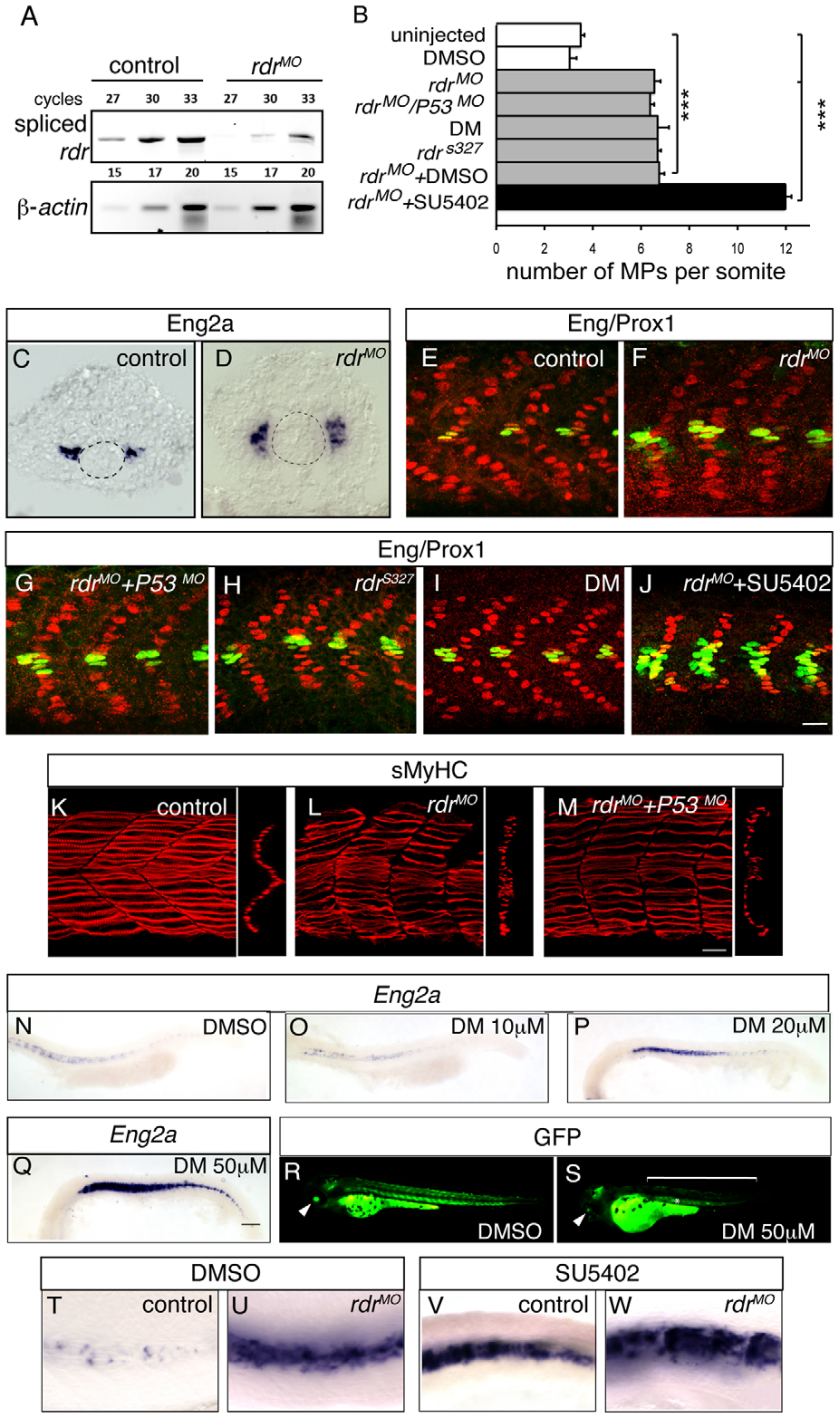
Radar or BMP signaling knock-down impairs MP formation. (A) The level of expression of spliced radar and β-actin mRNAs was analysed by semi-quantitative RT-PCR. Total RNA extracts were prepared at 1 dpf from uninjected embryos and from embryos that were injected with morpholinos blocking the splicing of radar (*rdr^MO^*). The number of cycles is shown. (B) Graphic representation of the number of MPs per somite at 1 dpf in the indicated conditions, values = mean, error bars = s.e.m., ***p<0.001. (C–D) *eng2a* expression at the 12-somite stage in (C) uninjected, or (D) *rdr^MO^*-injected embryos, as determined by ISH. (E–J) MPs staining with anti-Eng (green) and anti-Prox1 (red) antibodies at 1 dpf embryos in (E) uninjected, (F) *rdr^MO^*-injected embryos, (G) *p53^M^* ^O^- and *rdr^MO^*-injected embryos, (H) radar mutant (*rdr^s327^*), (I) Dorsomorphin (DM) treated embryos and (J) SU5402-treated embryos that were injected with *rdr^MO^*, scale bar = 20 µm. (K–M) sMyhc (red) expression in the somites of (K) WT, (L) radar morphans and (M) P53/radar double morphans (rdr^MO^/P53^MO^) at 1 dpf, 3D reconstructions of multiple confocal scans, lateral and cross section views, scale bar = 20 µm. (N–Q) *Eng2a* expression in the somites of 1 dpf embryos after treatment with increasing doses of Dorsomorphin (10, 20 and 50 µM) or with DMSO (1%), scale bar 100 µm. (R–S) GFP expression in Tg(5XBRE[vent2]:-20lid3:GFP) embryos at 60 hpf after (S) DM (50 µM) or (R) DMSO (1%) treatments, showing that DM treatment inhibits BMP signaling. (T–W) *Eng2a* expression in 1 dpf embryos that were (U–W) injected or (T–V) not with *rdr^MO^* and treated with either (T–U) DMSO or (V–W) SU5402.

Although the regional inhibition of FGF signaling can explain the localization of the MP precursors to anterior adaxial cells, it cannot explain the positioning of these progenitors to the DV midline of the somite. Several recent studies have shown that manipulation of BMP signaling can alter MP number [21], [22] and these studies also show that Smad5, a downstream effector of BMP signaling is activated in the dorsal and ventral adaxial cells and but not within cells of the central region of the compartment [22], [23]. Furthermore, Smad binding sites have been shown to regulate activity of the *eng2a* promoter [22], [23]. This has led to the suggestion that BMP activity could influence the fate of the myotome along the DV axis, although direct evidence for this assertion is lacking. Furthermore, Smad5 is also known to be activated by the TGF-ß signaling pathway in many biological systems and a number of *tgf-ß* genes are expressed during zebrafish during myogenesis complicating interpretation of these data [37]. To visualize BMP signaling more specifically we generated a transgenic line that expresses GFP under the control of a BMP Responsive Element which contains 5 tandem BRE elements derived from the Xenopus *vent2* gene coupled to a minimal Xenopus *id3* promoter, promoter elements known to specifically respond to BMP signal transduction. The activation of this transgene (Tg(5X*BRE[vent2]:-20lid3:GFP*) has been shown to occur specifically via the BMP signaling pathway, and not by other TGF-ß-related ligands [38], [39], [40] Figure 6A–6D). The expression of GFP in Tg(5X*BRE[vent2]:-20lid3:GFP*) embryos correlates with the distribution of phospho-Smad5 (Figure 6F). By early somitogenesis, BMP signaling is activated in the adaxial cells specifically in cells of the dorsal and ventral edges of the myotome, and reporter expression decreases in the midline (*n* = 14/14, Figure 6A, 6B, 6D, 6H) where MP precursor formation occurs (Figure 7C). Subsequent activity of the transgene is restricted to migrating adaxial cells but not to MPs (*n* = 12/12, Figure 6C and 6G). These data suggest that the different levels of BMP activation along the DV axis could control the dichotomy of the MP/SSFs cell fate choice.

As mentioned above, several BMP-like ligands are present in the tissues surrounding the myotome. *gdf6a*/*radar* exhibits polarized expression in the DV axis, with expression evident in the dorsal neural tube, hypochord, and the primitive gut endothelium [41], [42]. The specific temporal and spatial aspects of its expression suggest *radar/gdf6a* is the most likely BMP ligand to influence the DV patterning of the zebrafish myotome [41], [42]. To examine this question, we genetically down-regulated *radar/gdf6a* by the injection of antisense morpholinos specifically targeted to the zygotic *radar/gdf6a* mRNA (*rdr^MO^*, Figure 7A). Loss of zygotic *radar/gdf6a* function in Tg(*5XBre[vent2]:-201id3:gfp*) embryos causes a reduction of BMP activation evident within this line (*n* = 5, Figure 6E versus 6A), and a concomitant medio-ventral expansion of both the MP precursor domain (*n* = 6/6, Figure 7D versus 7C, Figure S5) and the number of differentiated MP cells at 24 hpf (*n_somite_* = 21, Figure 7B and 7F versus 7E, Figure S5) consistent with previously reported results [43]. To confirm the specific effect of the *rdr^MO^* we generated a *p53* and *radar* double morphant in which the number of MPs was similarly increased (Figure 7B and 7G) but non-Eng-positive SSFs now migrated properly at 24 hpf compared to the single radar morphant, (Figure 7M versus 7K, 7L). Furthermore, the phenotype of the *p53*/*rdr^MO^* injected embryos was identical to homozygous *rdr^s327^* mutant embryos [44] (*n_somite_* = 17, Figure 7B and 7H), an phenotype that could be reversed by careful titration with WT *rdr* mRNA injection (Figure S4). Embryos treated with Dorsomorphin (DM) (*n_somite_* = 17), a specific pharmacological inhibitor of BMP signaling [45], exhibited a dose dependent increase in MP number (Figure 7B and 7I, 7N–7Q) and a concomitant reduction of GFP expression in Tg(*5XBre[vent2]:-201id3:gfp*) embryos (Figure 7R, 7S and [22]). A similar increase in MP number is also seen when adaxial cells are cell autnomously inhibited from responding to BMP like ligands through use of a dominant negative form of the BMP receptor (*dnbmpr*) expressed from the adaxial specific *smyhc* promoter (*smyhc:dn-BMPr GFP*) (39.01% of transgenic fibres, n*_fibres_* = 326, Figure 4L and 4Q).

To elucidate whether FGF and BMP signaling co-operate to control adaxial cell fate, we examined the formation of MPs and SSFs when both pathways were simultaneously knocked down. *rdr* morpholino injections into SU5402-treated embryos caused an increase in MPs and *eng2a* expression compared to controls (DMSO, SU5402 treatment or *rdr^MO^* alone) that was essentially additive (*n_somite_* = 17, Figure 7B, 7J and 7T–7W), demonstrating that FGF and BMP cooperate to control the MP/SSF decision, and do so independently of one another.

### FGF and BMP signaling independently coordinate specification of adaxial cells in the AP and DV planes

**Figure 8:**
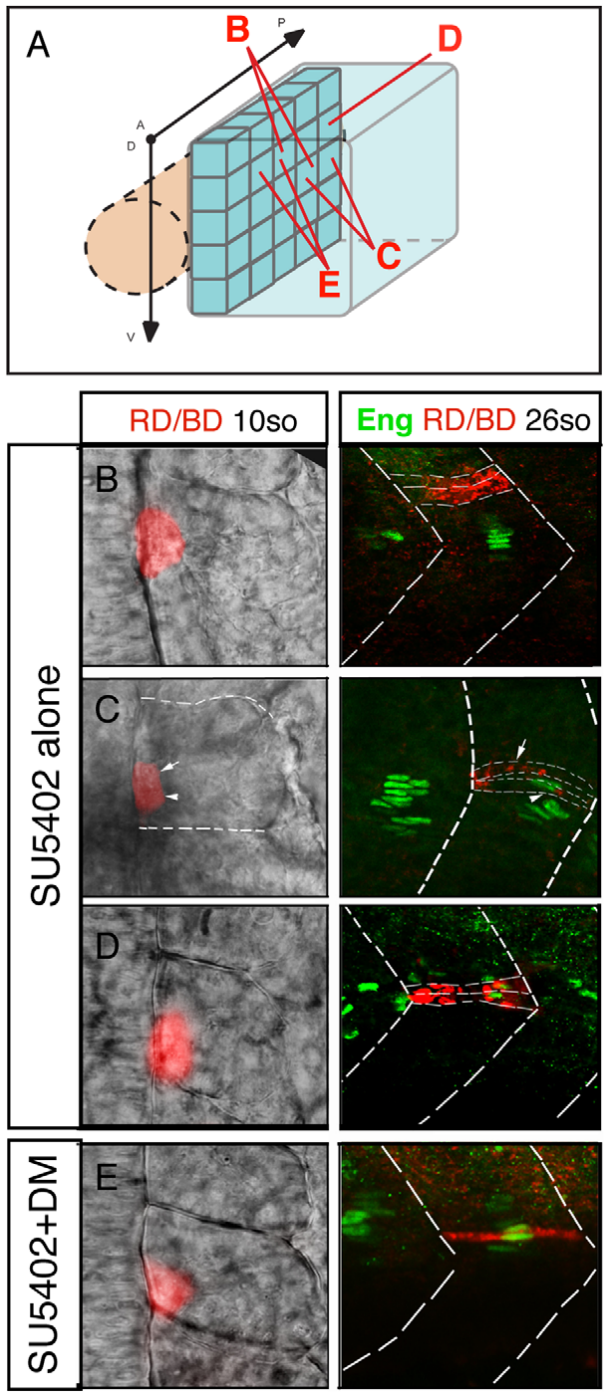
FGF and BMP signaling coordinates the fate of adaxial cells. (A) Individual adaxial cells located in the posterior region of the adaxial cells at various D/V positions were labeled according to the diagram. (B–C–D) The fate of the adaxial cells after treatment of SU5402 alone or (E) in combination with Dorsomorphin (DM) was followed using iontophoretic injections of TMRD (red) within the newly formed somites of 10- to 15-somite embryos (left panels, dorsal views). Their fate was analyzed using anti-Engrailed antibody staining (green) at 30 hpf (right panels, lateral views).

While the experiments outlined above, together with those of previously published studies, clearly show that BMP and FGF signaling can influence MP formation, they do not provide direct evidence for a role in DV or AP axis specification. It is possible that these signals could influence proliferation of MP precursors or recruitment to the adaxial cell compartment. In order to examine these issues more directly, we fate mapped the adaxial cell compartment using iontophoresis of TMRD into embryos where FGF signaling (SU5402 treatment) or FGF and BMP (SU5402+DM treatment) signaling had been inhibited (Figure 8A). According to our model, the MP domain should expand in the AP axis without FGF signaling and along both the AP and DV axes in the absence of either signal. Consistent with these predictions we found that MPs in SU5402-treated embryos could be derived from posterior adaxial cells (n = 8/12), a situation never observed in untreated embryos, but remained restricted to the mid-point of the DV axis (Figure 8B–8D). MPs in SU5402+DM-treated embryos arose from a pool of progenitors expanded in both the DV and AP axes of the adaxial cell equivalence group (n = 7/11, Figure 8E). Collectively, these results demonstrate that FGF and BMP signaling synergize to control specification of adaxial cells in the AP and DV axis, respectively.

## Discussion

### A fate map for the HH-dependent adaxial cell compartment

At the beginning of segmentation all adaxial cells are columnar shaped epithelial-like precursors that align medially along the notochord, and display no morphological asymmetry. By initially undertaking fate map analyses of the entire forming myotome we have defined the adaxial cell compartment as a cell fate equivalence group that gives rise to these two specific slow muscle cell fates, the MPs and the SSFs. We have further defined mechanistically how these precursors are induced to give rise to these two distinct populations. The adaxial cells differentiate asynchronously within newly formed somites, with the cells adjacent to the anterior somitic border and located at the mid-point of the DV axis of the somite being the first to initiate the morphogenetic and differentiation movements we have previously describe [9]. This morphogenetic asymmetry is mirrored at the molecular level where the same cells that undergo precocious differentiation simultaneously initiate expression of the MP specific marker gene *eng2a*. This analysis suggests that these cells are the progenitors of the MP cells. In order to examine this question directly we generated a fate map of the adaxial compartment and found that each slow muscle fiber type (SSF and MP) arose from a specific region of the adaxial cell array. While the anterior adaxial cells at the DV mid-point of the somite give rise to MP within the midline, the non-MP precursor adaxial cells go on to form the SSF palisade at the lateral surface of the myotome in direct topographical reflection of their position in the pre-migratory adaxial compartment. These data indicate that both dorso-ventral and the anterior-posterior identities need to be determined coordinately within the adaxial cell equivalence group for cell fate determination to occur correctly.

Previous analyses have indicated that HH signaling is required to specify the adaxial cells prior to the onset of segmentation and that levels of HH influence the fate of these cells [7], [14], [18]. However, in the absence of HH signaling, cells with a distinct morphology still form adjacent to the notochord, indicating that not all aspects of adaxial cell morphogenesis are controlled by HH signal transduction [7]. In the absence of HH signal activation, a fast twitch muscle gene expression profile is activated within these cells instead of genes indicative of the slow muscle lineage. Consequently, these cells differentiate as fast MyHC expressing, cells stochastically dispersed throughout the myotome [7]. Despite the ability of HH signaling to control the determination of the slow muscle fate, the three HH ligands expressed in the embryonic midline (*ehh*/*ihhb*, *shh*/*shha*, *twhh*/*shhb*, [12], [20], [46], [47] are not restricted in the anterior-posterior direction, nor is there any indication that HH target genes are asymmetrically activated within the nascent adaxial cell compartment in either the anterior-posterior or dorso-ventral planes. Furthermore, we could also find no variation in the length of the primary cilia in adaxial cells, in line with the lack of modulation of HH target gene expression within adaxial cells. Thus, a model involving distinct regulators of cell fate needed to be invoked in order to conceptually generate the MP fate from the anterior-most cells of the dorso-ventral midline of the adaxial cell equivalence group.

### A lack of FGF signaling in anterior adaxial cells induces MP fate

Many studies have examined the role of FGF signaling during myogenesis *in vitro*, where it has been shown to promote cell proliferation and represses myoblast differentiation. It has also been shown that early myoblast precursors require FGF in order to subsequently express their myogenic phenotype [48], [49]. However, despite these extensive *in vitro* studies the exact function of Fgf in the activation or the repression of muscle differentiation *in vivo* is controversial and appears to often to contradict this simple repressive role defined *in vitro* [50]. For example, zebrafish Fgf8-mediated signaling has been shown to drive the terminal differentiation of fast-twitch but not slow-twitch muscle fibers, and simultaneously also controls proliferation of the external cell progenitor layer, the equivalent of the amniote dermyotome [25], [51]. In amniote embryos, FGF signaling has been implicated in myogenesis *in vivo*, both in promoting progenitor cell proliferation [52] and in promoting their differentiation [53], [54]. In chick embryos most, if not all, replicating myoblasts present within the skeletal muscle masses of the limb express high levels of the FGF receptor FREK/FGFR4 and the inhibition of FgfR4 leads to a dramatic loss of limb muscle [54], [55]. Conversely, over expression of FGF in the chick somite leads to muscle differentiation suggesting that, as in the zebrafish lateral myotome, myogenic differentiation is positively controlled by FGF signaling [25], [54]. This is consistent with observations in mouse where ectopic expression of the cell autonomous negative regulator of FGF signaling *sprouty2* in myogenic progenitors inhibits their differentiation [53].

Here we show that the FGF pathway does play a role in muscle formation but it is downstream of the HH dependent process of slow-twitch fiber specification. FGF signaling is asymmetrically activated in the adaxial cells. Specifically, within anterior adaxial cells it is strongly reduced, to the point of complete inhibition of specific FGF target genes. We have shown, using a combination of genetic and pharmacological approaches that down regulation of the FGF pathway promotes MP formation at the expense of SSFs within the adaxial cell compartment. This does not appear to be driven by the restriction of the expression of FGF ligands, since the FGF encoding genes, Fgf8a and Fgf17, are both localized to the anterior somite [25]. Rather, FGF signaling in anterior adaxial cells is inhibited by a cell autonomous negative regulator of the FGF signaling cascade, *spry4* [30]. *spry4* expression is induced by FGF signaling and has been shown to act in a negative feed back loop on the FGF pathway in a number of contexts (this present study and [27], [31]). The direct role of *spry4* in MP formation is demonstrated by data that shows that the ectopic expression of *spry4* in the adaxial cells induces MPs while its inactivation in *spry4* mutant embryos inhibits this fate. Therefore, our results suggest a model where *spry4* is activated within the anterior adaxial cell compartment in response to high levels of adjacent FGF ligands that ultimately suppress FGF signaling within these cells, thereby breaking equivalence in the anterior posterior dimension. This role appears to be more analogous to that played by FGF signaling during organogenesis rather than those outlined above for myogenesis, where the fate of various stem and progenitor cells are partitioned by activation or inhibition of FGF signaling in organs as diverse as the liver and pancreas [56], ear [57], [58] and teeth [59] often in conjunction with opposing cell fate determining signals, including BMP signaling.

### BMP signaling determines dorso-ventral identity of the adaxial cell equivalence group

While FGF signaling restricts the fate of the adaxial cells to the anterior most cells of the myotome, a second signal is needed to restrict the positioning of these cells in the dorso-ventral dimension. Recently, studies have demonstrated that the downstream effector of BMP signaling, p-Smad5 is specifically restricted to the dorsal and ventral adaxial cells, and is absent from cells of the dorso-ventral midline of the myotome [22], [23]. Furthermore, several previous studies have shown that manipulation of BMP signaling can influence the number of engrailed positive MPs [21], [43]. Indeed, the ectopic expression of chick Dorsalin-1, a BMP-like family member, in the zebrafish notochord inhibits MP development [21]. More recent studies have shown that inhibition of BMP via use of the small molecule inhibitor Dorsomorphin, or morpholinos against the BMP receptor *bmpr1ba*, results in an increase of MPs [22], [23]. However, exactly how BMP influences the formation of these muscle subtypes has remained unclear. Here we show that the fate of the adaxial cells is specified in the DV axis by a radar-mediated BMP signaling. This statement is supported by several lines of evidence. Firstly, a transgenic reporter line specific for BMP signaling reveals that at the onset of segmentation, BMP signaling is active in the dorsal and ventral most adaxial cells, but absent from in the DV mid-point of the forming myotome. This region of low BMP activity of correlates with the location of MP precursor specification, as specifically determined via our fate map analysis. Secondly, BMP signaling is mediated by *gdf6a/radar* in the adaxial cells and knockdown of BMP activity modifies the fate muscle precursors in the adaxial compartment and promotes MP formation in a dose dependant manner.

Previous analysis of the activity of BMP signaling during muscle formation in amniotes has provided evidence that it negative regulates the myogenic program [60], [61] a role it appears to also play in controlling the proliferation and the onset of myogenesis within the external progenitor cell layer of the zebrafish myotome [62]. However, in the context of the adaxial cells it does not appear to influence the proliferation of these progenitors, the timing of entry of these cells into myogenesis or the differentiation of the adaxial cells themselves. Our lineage analysis specifically illustrates that it alters the fate of this progenitor compartment.

### The activities of the HH, FGF, and BMP signaling pathways specify MP identity

In this study we show that in contrast to HH signal transduction, FGF and BMP signaling has no effect on the slow muscle fate but instead regulates the decision of adaxial cell progenitors to become either SSF or MP cells. Indeed, as discussed above, the activation of these signaling pathways promotes SSF formation while their decrease or absence promotes MP formation. Modulation of FGF or BMP signals does not affect HH signaling and the consequences of their knockdown on the adaxial fate are additive (this study and [22]). Similarly, manipulation of the level of HH signaling (mutants within the HH pathway or cyclopamine treatment) does not affect the expression pattern of phospho-Smad 5, suggesting that HH signaling does not influence cell fate indirectly through BMP signaling [22], [23]. Thus the FGF and BMP signals act independently of, and synergistically with, each other to control the SSF/MP cell fate dichotomy. Intriguingly, the application of both FGF and BMP is required for the induction of a specific muscle cell fate, the Pax7-positive satellite cell progenitors, in Xenopus animal caps [63]. This suggests that the synergistic action of BMP and FGF may operate to specify other muscle cell types.

While HH and BMP signalling have been demonstrated to coordinate cell fate determination in the chick neural tube [64] and HH, BMP and FGF signalling collectively control the specification of numerous cell types in vertebrate and invertebrate systems, the majority of these studies do not examine the fate of individual cells in real time. The developmental paradigm of the adaxial cells allows single cells to be labelled and tracked and their fate determined within a genetically defined cellular equivalence group in the living animal a set of attributes that is to our knowledge unique in vertebrate developmental systems. We therefore believe that our study suggests that the adaxial of zebrafish could emerge as a paradigmatic example of a vertebrate cell fate equivalence group, in the same manner as the, *Drosophila* neuroectoderm, parasegment and imaginal disc and the *C. elegans* vulva [1]–[6] which have provided exquisite cellular and genetic resolution to generate a detailed understanding of cell specification mechanisms within invertebrate systems.

**Figure 9:**
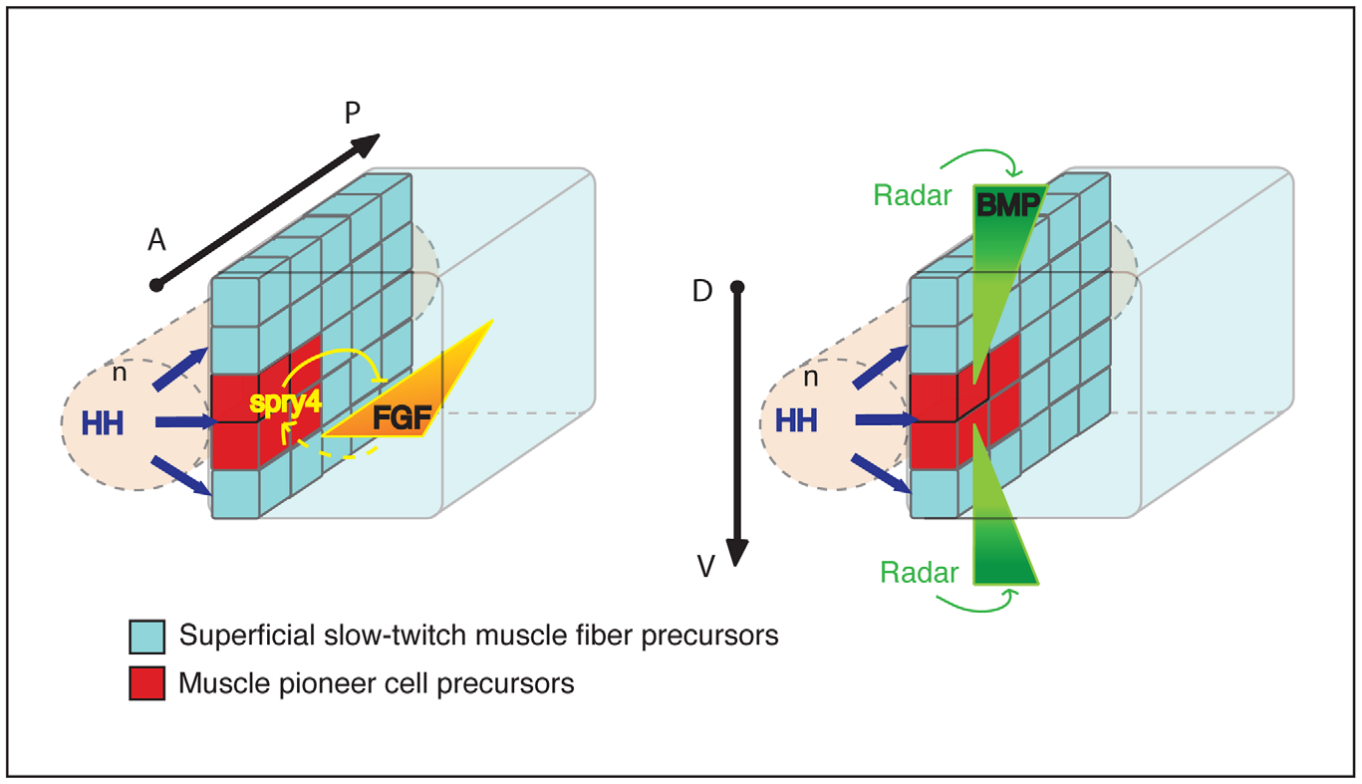
Model of the synergic action of FGF and BMP signaling on adaxial cell specification. The diagram represents the adaxial compartment in the zebrafish somite, adjacent to the notochord (n), at the beginning of segmentation. The anterior-posterior (A/P) and dorsal-dentral (D/V) axes are shown. The adaxial cell pool is initially specified by HH signal transduction from the embryonic axial structure. Spry4-mediated regional inhibition of FGF restricts the MP fate to the most anterior adaxial cells. By contrast, the absence of Radar-mediated-BMP signaling at the dorso-ventral mid-point of the somite restricts MP fate at the dorso-ventral mid-point of the myotome. The combination of the three signals forms a 3-D patterning system that coordinates the specification of adaxial cells into muscle pioneer cells and superficial slow fibers.

Our results also demonstrate an integrated signaling milieu that coordinates the specification of muscle cell fates within the adaxial cell compartment. The adaxial cell pool is initially specified in the somitic region adjacent to the notochord by HH signal transduction from the embryonic midline. This, together with regional inhibition of FGF in the anterior-most adaxial cells and a lack of BMP signaling at the DV midpoint of the somite, creates a 3-Dimensional network of signals that restricts the MP fate to the most anterior cells within a specific cellular equivalence group in the developing myotome (Figure 9). These signals act independently from each other to determine fate and uniquely MP specification is controlled by the action of different signal transduction pathways that act specifically to direct specification in distinct axial dimensions. This essentially Cartesian system of cell fate determination is somewhat reminiscent of that deployed during the development of the ventral nerve chord of *Drosophila* where a complex series of patterning genes are deployed in gradients along the DV and AP axes to induced specific fate determining genes within individual neuroblasts within the neuroectodermal sheet [3], [4], [5]. However, in the case of the adaxial cells there is no evidence for a role of lateral inhibition, which in the *Drosophila* ventral neuroectoderm is required for the expression of individual proneural genes and adoption of specific fates [4], [5]. Furthermore, our results reveal that individual secreted signals act in specific dimensions within this Cartesian system, rather than in a cooperative or mutually exclusive manner to specify cell fate, the prevalent ways by which cells are determined in vertebrate systems.

## Materials and Methods

### Zebrafish line and maintenance

Fish maintenance, staging and husbandry were as described previously [65]. Wild-type embryos of the TE strain were used in all staining and manipulation. Mutant alleles used were *spry4^fh117^* (ZIRC, direct submission from the laboratory of Cecila Moens), *radar/gdf6a^s327^* (kind gift of Herwig Baier). Transgenic lines used were *Tg (hsp70:dnFgfr1-EGFP)^pd1^* and Tg(*5XBre[vent2]:-201id3:gfp*).

### *In situ* hybridization, antibody staining, sectioning, microscopy, and statistical analysis

*In situ* hybridization, antibody staining, and microtome sectioning were performed as previously described [65]. Probes were obtained by PCR amplification or from existing clones: *sprouty4*, *erm* (cb805), *pea3* (IRBOp991G0430D) and *eng2a* [65]. *In situ* hybridizations on whole mount embryos were performed using digoxigenin (DIG)-labeled (Roche) antisense RNA probes and nitro blue tetrazolium/5-bromo-4-chloro-3-indolyl phosphate (NBT/BCIP) or fast red (Sigma). Microtome sectioning was performed on ISH stained embryos. Antibodies used were: anti-sMyHC (1/10, F59, DSHB Iowa, USA), anti-GFP (1/500, Rockland), anti-Engrailed (1/10, 4D9, DSHB Iowa, USA), anti-Prox1 (1/150, Fitzgerald), anti-Phospho-Smad5 (1/100, Cell signaling technology), anti-diphospho-ERK (1/10000, Sigma) streptavidin-alexa546 (1/1000, Molecular Probe). Vibratome sectioning was performed before antibody staining when necessary. 3D reconstructions were performed using Nikon C1 and Leica SP5 Confocal microscopes and Imaris software.

### Statistical analysis

Counts of the number of differentiated MPs or SSFs were performed in the yolk extension region of 6 to 15 embryos. Analysis of variance (ANOVA) determined statistical significance of differences within a 95% confidence interval. In specific figures the following statistics were applied: Figure 4L: ANOVA analysis, Figure 5K: ANOVA analysis, Figure 7B: ANOVA analysis, Figures S1, S3, S5: Student test, 2 tails, unpaired, Table S1: ANOVA analysis.

### Assembly of DNA constructs and RNA for live imaging

All constructs were assembled from entry clones using the Tol2kit (Kwan et al 2007). For transcription of RNA for whole-somite imaging, we assembled CMV/SP6-EGFPcaax and CMV/SP6-H2/afz-mCherry. Plasmids were linearized with NotI before transcription of capped RNA using an mMessage-mMachine kit (Ambion). Vectors used for mosaic analysis of single cells were *smyhc1:spry4-IRES-EGFP, smyhc1:EGFP, smyhc1:dnBMPr GFP and smyhc1:dnspry4-IRES-EGFP*. The new entry clone p5E-smyhc1 was made by subcloning the *smyhc1* promoter from the plasmid *p9.7kbsmyhc1:GFP-I-SceI* (Elworthy et al 2008) into p5E-MCS (Kwan et al 2007). The pME-*spry4* clone was made by cloning the full-length *spry4* ORF into pDONR221. Similarly, the ORF of Xenopus type Ia BMPr truncated in C terminal (BMPrΔC) from BMPR22 construct ([66] or of dominant negative form of *spry4* (spry4Y52A) from the pCS2-spry4Y52A were also cloned into pDONR221.

### Injections, drug treatments, and heat shock inductions

Injections were performed as described previously [65]. 40 ng/µl of DNA encoding *smyhc:spry4 ires GFP* or *smyhc:GFP* were injected in one cell stage. Adaxial cells were imaged in embryos where 25 ng/µl of both *CAAX-GFP* and NLS-*mCherry* encoding mRNAs were injected at the one cell stage. 3 ng/µl of *radar* morpholino alone (5′-GCAATACAAACCTTTTCCCTTGTCC-3′) or in combination with 3 ng/µl of *p53* morpholinos (5′-GCGCCATTGCTTTGCAAGAATTG-3′) were injected at the once cell stage. SU5402 (calbiochem) was added to the embryo medium at gastrulation or between 6- to 10-somites at a final concentration of 80 µM and maintained until the appropriate stage. 10 to 50 µM Dorsomorphin (Sigma) was applied to similarly staged embryos. Heat shock induction of *dn-fgfr1* expression was carried out at 6-somite stage. *(hsp70:dnFgfr1-EGFP)^pd1^* transgenic embryos in there plate were placed at 38° during 2 hours. GFP expression was visualized immediately after heat shock to confirm the expression of the transgenic protein.

### Iontophoresis injections

Iontophoresis injections as described in [67] with the following modifications: rhodamine dextran (10,000 MW, Molecular Probes, 5 mg/ml) combined with Biotin dextran (10,000 MW, Molecular Probes, 1.5 mg/ml) were injected into cells of agarose-imbedded, 10- to 15-somite stage embryos. Adaxial cell labelings were positioned on the dorso-ventral axis via references to adjacent tissue landmarks within injected embryos and were imaged as previously described. The labeled embryo was dissected free of agarose and was allowed to develop until 30 hpf; it was then remounted in a 3% solution of methylcellulose (Sigma) and imaged. Subsequently, the embryo was fixed 2H in 4% paraformaldehyde and sequentially stained for Engrailed and sMyhc as described above.

## Supporting Information

Figure S1 FGF inhibition does not alter the number of medial fast fibers. (A) Prox1 (red) and Engrailed (green) expression in the somites of 1 dpf WT embryos after DMSO or SU5402 treatments. SU5402-mediated FGF inhibition does not change the number of medial fast fibers (MFF), here revealed by a low expression of engrailed but not Prox1 expression (*). (B) Graphic representation of the number of MFF per somite of 1 dpf WT embryos after DMSO and SU5402 treatments, values = means, error bars = standard error of the mean (s.e.m.). The difference between the two conditions is not significant (NS, p value = 0.34).
(TIF)

Figure S2 Hedgehog and primary cilia are not affected by FGF signaling inhibition. (A) *ptc1* expression is similar in DMSO and in SU5402 treated embryos at 13-somite, as determined by in situ hybridization. Flat mounted embryos, dorsal view, anterior towards the top. (B) Acetylated-tubulin and γ-tubulin expression in 13-somite embryos after DMSO or SU5402 treatment. Number and length of primary cilia of the adaxial cells in the presomitic mesoderm (arrow heads) are unaffected by SU5402 treatment. Pictures are single confocal scans, dorsal view. Dashed line shows the limit between the notochord (nc) and the presomitic mesoderm. Adaxial cells are adjacent to the notochord. Scale bars: 10 µm.
(TIF)

Figure S3 Relative distance between MP nuclei and anterior boundaries of the somites. Distance between highly Engrailed expressing cells and anterior boundaries of the somites was measured using confocal microscopy and ImageJ software.
(TIF)

Figure S4 Radar/gdf6a morpholinos-induced phenotype is reversed by WT rdr mRNA injection. (A–E) Live embryos at 1 dpf either (A) uninjected or (B) injected with rdr morpholinos (*rdr^MO^*) showing reduced eyes, massive cell death in the anterior neural tube and flatten somites (arrow heads, C1 phenotype). (C–D) Injections of 10–100 pg of *rdr* mRNA either alone (not shown) or in combination with *rdr* ^MO^ induce severe gastrulation defects as described in Sidi et al, 2003 (C2 and C3 phenotype). (E) Only a low quantity of *rdr* mRNA (0.5–1 pg) rescue the phenotype induced by *rdr* ^MO^ injection (C4 phenotype or WT). (F) Graphic representation of the percentage of embryos exhibiting WT, C1–C4 or dead phenotype in indicated conditions. (G–I) Prox1 (red) and Engrailed (green) expression in the somites of 1 dpf embryos either (G) uninjected or injected with (H) *rdr* ^MO^ alone or (I) in combination with 1 pg of *rdr* mRNA. Increased MP number observed in *rdr* ^MO^ injected embryos is reversed by WT *rdr* mRNA, showing that altered MP number is specifically induced by *rdr* loss-of-function.
(TIF)

Figure S5 Supernumerary MPs in rdr morphans are mainly localised in the midline and in the ventral region of the myotome. MPs were stained with anti-Eng (green) and prox1 (red) antibodies in uninjected controls (A) and rdr MO injected (B) embryos at 1 dpf. The midline (solid line), midline region (dashed lines), ventral and dorsal regions are shown. Here the midline region corresponds to the 5 µm regions flanking either side of the midline. Scale bar = 25 µm (C) Graphic representation of the number of MPs in the midline, dorsal and ventral regions of the somite in uninjected controls and rdr morphans. ***p<0.001 and **p<0.005, values = means and error bars = S.E.M.
(TIF)

Table S1 Manipulation of FGF and/or BMP signaling pathways does not affect slow-twitch lineage specification. The table represents the number of slow muscle cells per somite. These cells were counted using the expression of sMyHC or Prox1 in the yolk extension region. Values represent the means ± standard error of the mean (s.e.m) and the total number of somites counted for the experiment. Analysis of variance (ANOVA) shows no statistical difference within a 95% confidence interval between the treatments/genotypes.
(DOC)

Video S1 Anterior adaxial cells differentiate and elongate first. This movie shows the adaxial cells behaviour occurring in their very first differentiation phase. Embryos were labelled with a membrane-bound GFP (green) and a nuclear localised mCherry (red) and imaged in a continuous 4D time-lapse analysis that covers a period of 30 minutes. The first part of the movie corresponds to a dorsal view in the dorso-ventral midline focal plan between 0 min to 30 min. The second part is an overview of the focal plans above and below the DV mid-point at 30 min. This movie reveals that the anterior most-adaxial cell in the dorso-ventral midline is the first to differentiate. Adaxial cells above and below are less differentiated.
(MOV)

Video S2 Anterior adaxial cells are non migratory. This movie shows adaxial cell behaviour during the migration period (15–22 somite) captured using fluorescence microscopy. Embryos were injected at 1-cell stage with a DNA construct containing the GFP gene under the control of the *smyhc* promoter to induce mosaically the expression of the GFP in individual the adaxial cells. Here the transgenically labelled, partially elongaet, adaxial cell located in the anterior most position of the somite at its DV mid point (*) remain adjacent to the notochord. Cells in the more anterior somites are actively migrating to the lateral surface of the myotome, and quickly move out of focus as the movies progresses. Anterior (A) and posterior (P) orientation, notochord (nc) and somite boundaries (arrows) are shown. (dorsal view, anterior toward the bottom left). (AVI). Frames every 20 mins, 450 mins total.
(AVI)
