# Supplementary figures and images for: Morphogenesis and Cell Fate Determination within the Adaxial Cell Equivalence Group of the Zebrafish Myotome
Source: PLoS Genet. 2012 Oct 25;8(10):e1003014. doi: 10.1371/journal.pgen.1003014 (PMC3486873; doi:10.1371/journal.pgen.1003014)

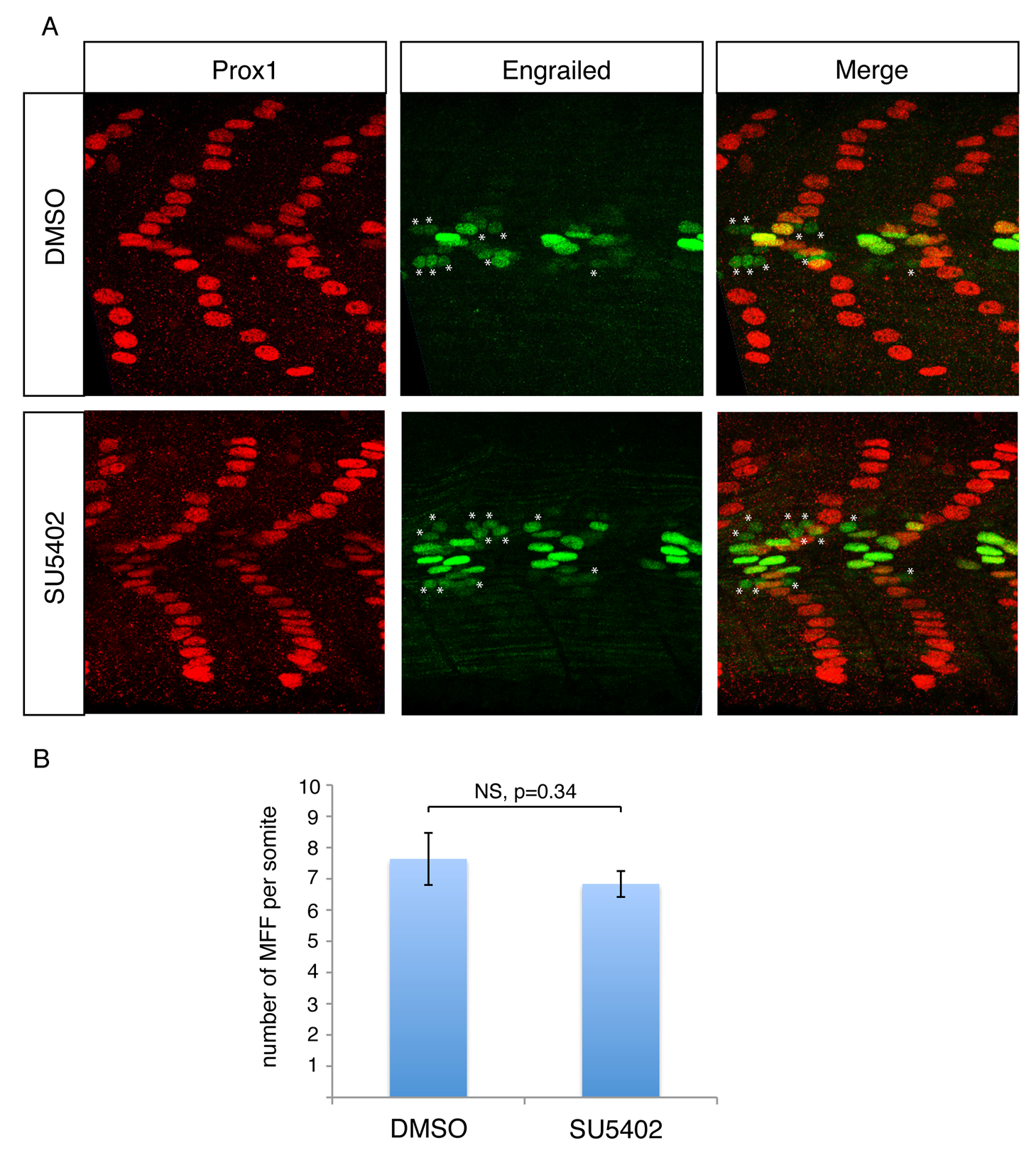

Supplement: Figure S1 — FGF inhibition does not alter the number of medial fast fibers. (A) Prox1 (red) and Engrailed (green) expression in the somites of 1 dpf WT embryos after DMSO or SU5402 treatments. SU5402-mediated FGF inhibition does not change the number of medial fast fibers (MFF), here revealed by a low expression of engrailed but not Prox1 expression (*). (B) Graphic representation of the number of MFF per somite of 1 dpf WT embryos after DMSO and SU5402 treatments, values = means, error bars = standard error of the mean (s.e.m.). The difference between the two conditions is not significant (NS, p value = 0.34). (TIF) [file pgen.1003014.s001.tif]

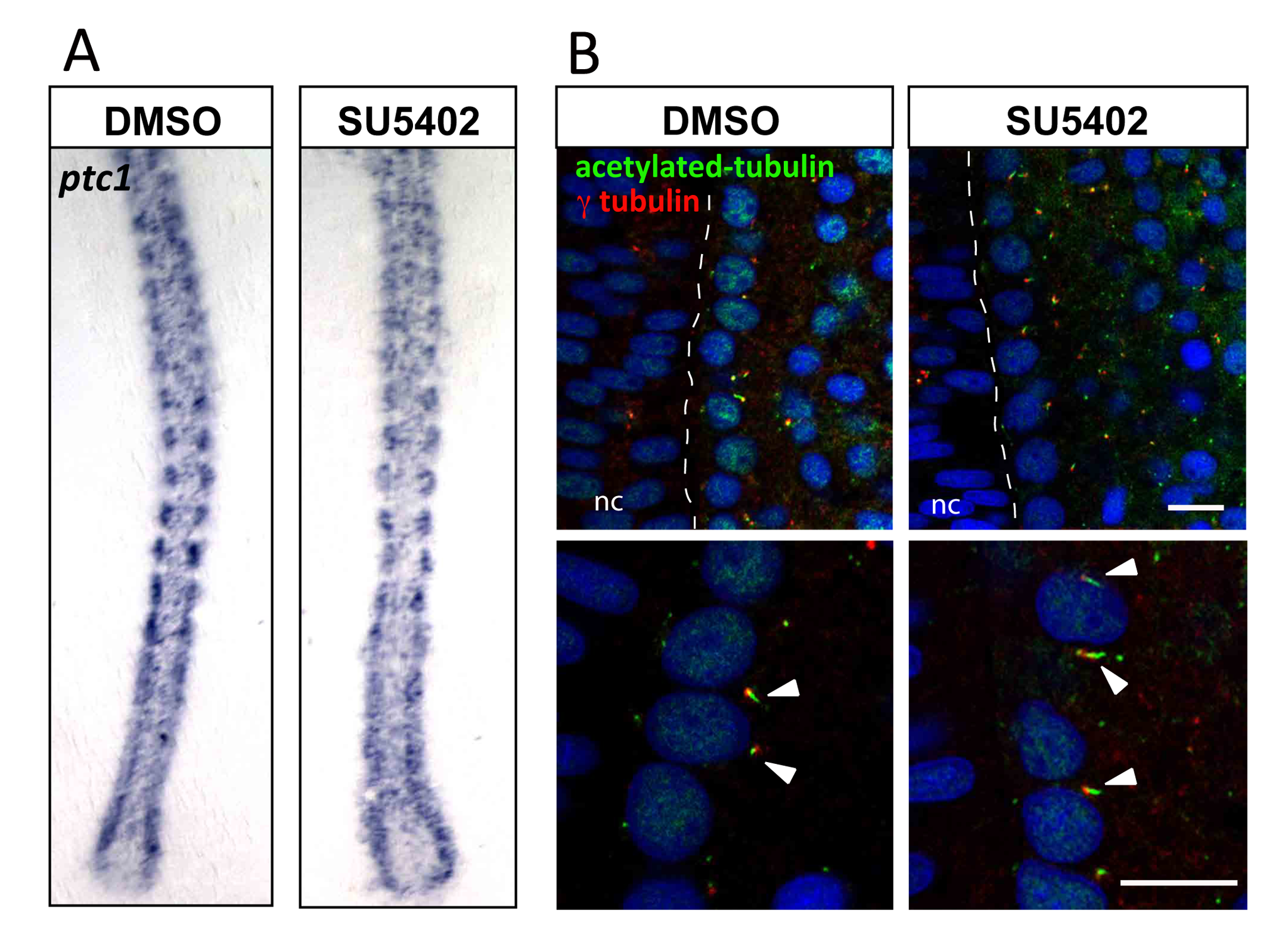

Supplement: Figure S2 — Hedgehog and primary cilia are not affected by FGF signaling inhibition. (A) ptc1 expression is similar in DMSO and in SU5402 treated embryos at 13-somite, as determined by in situ hybridization. Flat mounted embryos, dorsal view, anterior towards the top. (B) Acetylated-tubulin and γ-tubulin expression in 13-somite embryos after DMSO or SU5402 treatment. Number and length of primary cilia of the adaxial cells in the presomitic mesoderm (arrow heads) are unaffected by SU5402 treatment. Pictures are single confocal scans, dorsal view. Dashed line shows the limit between the notochord (nc) and the presomitic mesoderm. Adaxial cells are adjacent to the notochord. Scale bars: 10 µm. (TIF) [file pgen.1003014.s002.tif]

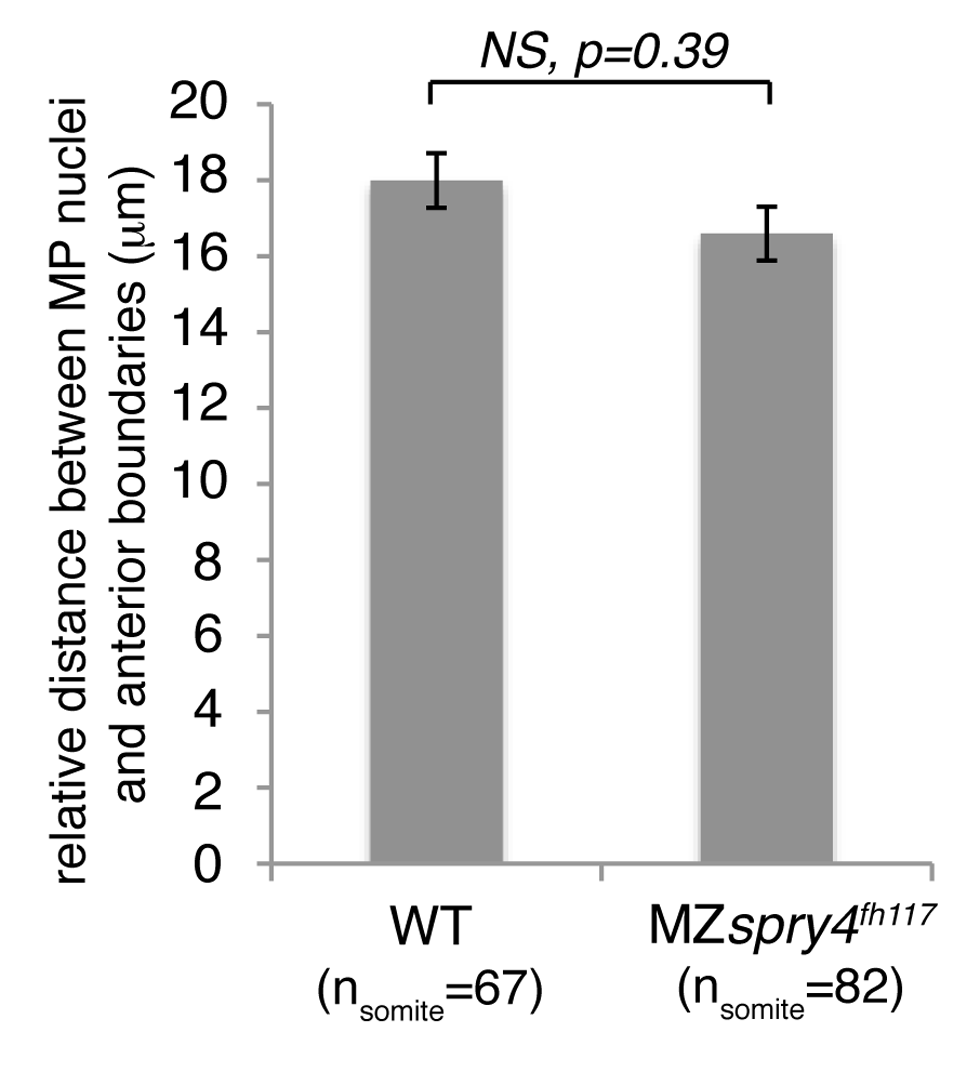

Supplement: Figure S3 — Relative distance between MP nuclei and anterior boundaries of the somites. Distance between highly Engrailed expressing cells and anterior boundaries of the somites was measured using confocal microscopy and ImageJ software. (TIF) [file pgen.1003014.s003.tif]

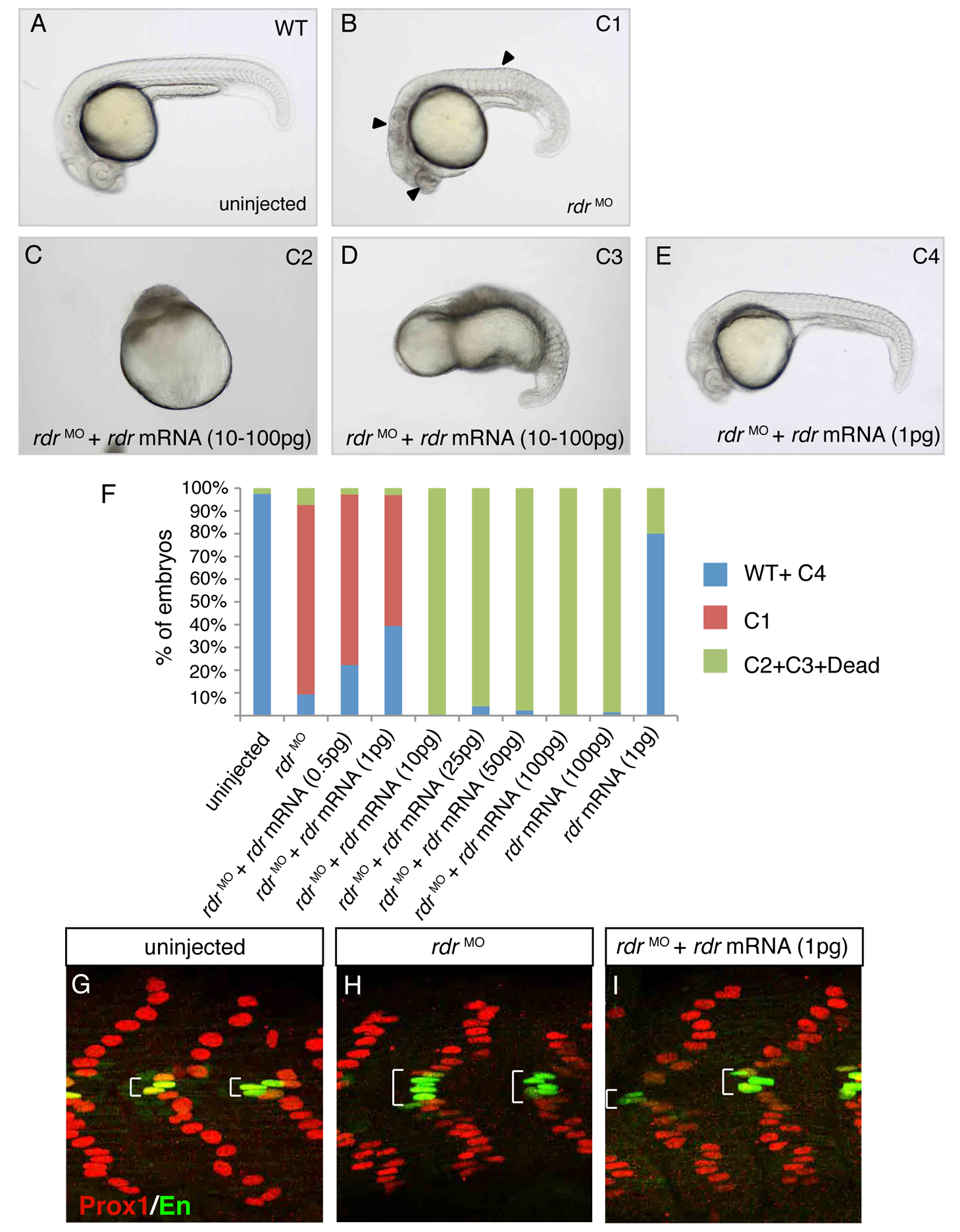

Supplement: Figure S4 — Radar/gdf6a morpholinos-induced phenotype is reversed by WT rdr mRNA injection. (A–E) Live embryos at 1 dpf either (A) uninjected or (B) injected with rdr morpholinos (rdrMO) showing reduced eyes, massive cell death in the anterior neural tube and flatten somites (arrow heads, C1 phenotype). (C–D) Injections of 10–100 pg of rdr mRNA either alone (not shown) or in combination with rdr MO induce severe gastrulation defects as described in Sidi et al, 2003 (C2 and C3 phenotype). (E) Only a low quantity of rdr mRNA (0.5–1 pg) rescue the phenotype induced by rdr MO injection (C4 phenotype or WT). (F) Graphic representation of the percentage of embryos exhibiting WT, C1–C4 or dead phenotype in indicated conditions. (G–I) Prox1 (red) and Engrailed (green) expression in the somites of 1 dpf embryos either (G) uninjected or injected with (H) rdr MO alone or (I) in combination with 1 pg of rdr mRNA. Increased MP number observed in rdr MO injected embryos is reversed by WT rdr mRNA, showing that altered MP number is specifically induced by rdr loss-of-function. (TIF) [file pgen.1003014.s004.tif]

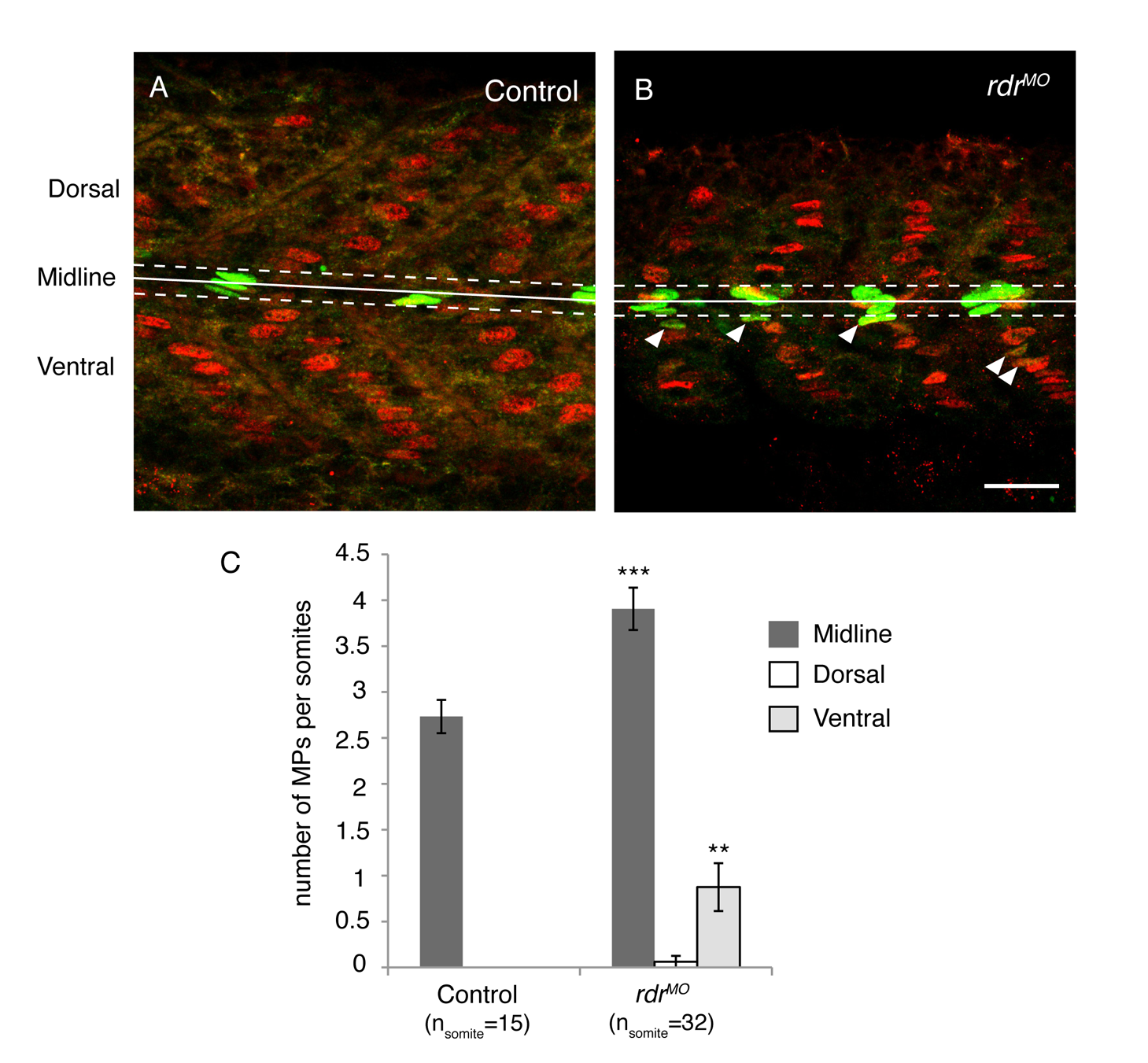

Supplement: Figure S5 — Supernumerary MPs in rdr morphans are mainly localised in the midline and in the ventral region of the myotome. MPs were stained with anti-Eng (green) and prox1 (red) antibodies in uninjected controls (A) and rdr MO injected (B) embryos at 1 dpf. The midline (solid line), midline region (dashed lines), ventral and dorsal regions are shown. Here the midline region corresponds to the 5 µm regions flanking either side of the midline. Scale bar = 25 µm (C) Graphic representation of the number of MPs in the midline, dorsal and ventral regions of the somite in uninjected controls and rdr morphans. ***p<0.001 and **p<0.005, values = means and error bars = S.E.M. (TIF) [file pgen.1003014.s005.tif]
