# Supplementary material for: Morphogenesis and Cell Fate Determination within the Adaxial Cell Equivalence Group of the Zebrafish Myotome
Source: PLoS Genet. 2012 Oct 25;8(10):e1003014. doi: 10.1371/journal.pgen.1003014 (PMC3486873; doi:10.1371/journal.pgen.1003014)
Supplement: Table S1 — Manipulation of FGF and/or BMP signaling pathways does not affect slow-twitch lineage specification. The table represents the number of slow muscle cells per somite. These cells were counted using the expression of sMyHC or Prox1 in the yolk extension region. Values represent the means ± standard error of the mean (s.e.m) and the total number of somites counted for the experiment. Analysis of variance (ANOVA) shows no statistical difference within a 95% confidence interval between the treatments/genotypes. (DOC) [file pgen.1003014.s006.doc]

**Supplemental Table 1: Manipulation of FGF and/or BMP signaling pathways does not affect slow-twitch lineage specification.**

The table represents the number of slow muscle cells per somite. These cells were counted using the expression of sMyHC or Prox1 in the yolk extension region. Values represent the means ± standard error of the mean (s.e.m) and the total number of somites counted for the experiment. Analysis of variance (ANOVA) shows no statistical difference within a 95% confidence interval between the treatments/genotypes.
